# Prussian Blue Analogues for Non‐Aqueous Sodium‐Ion and Potassium‐Ion Batteries: The Landscape From Lab‐Scale Optimizations Toward Practical Applications

**DOI:** 10.1002/smll.202514168

**Published:** 2026-01-27

**Authors:** Yingkangzi Mei, Charlie A. F. Nason, Yang Xu

**Affiliations:** ^1^ Department of Chemistry University College London London UK

**Keywords:** full cell, practical use, Prussian blue analogues, structure–performance relationship, sustainable energy storage

## Abstract

Prussian blue analogues (PBAs) have emerged as promising cathode materials for next‐generation potassium‐ion (PIBs) and sodium‐ion batteries (SIBs) owing to their simple synthesis, low cost, structural robustness, and well‐balanced electrochemical properties. Despite these advantages, several intrinsic challenges continue to limit their practical implementation. This review provides a concise roadmap connecting material‐level fundamentals with key lab‐scale optimization strategies, and highlights the factors that most critically influence the real‐world viability of PBAs. Beyond targeting high performance, we evaluate the practicality of different approaches, with particular attention to particle size, crystal water, and safety considerations. The discussion aims to guide researchers in advancing PBAs toward scalable non‐aqueous energy‐storage systems and in supporting the broader development of sustainable battery technologies.

## Graphical abstract

Prussian blue analogues (PBAs) are scalable cathode materials for non‐aqueous sodium‐ (SIBs) and potassium‐ion batteries (PIBs). This review links crystal chemistry, defect control, and particle engineering with electrochemical performance, highlighting how synthesis, crystal water, and interfacial stability jointly determine practical energy density, safety, and cycle life in next‐generation sustainable battery systems.

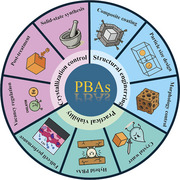

## 1 Introduction

Potassium and sodium, the sixth and seventh most abundant elements in the Earth's crust, have become attractive candidates for next‑generation energy‑storage systems. Their low cost, environmental friendliness, and the shared “rocking‑chair” mechanism with commercial lithium‑ion batteries (LIBs) allow partial transfer of existing battery knowledge and manufacturing. Potassium‑ion (PIBs) and sodium‑ion batteries (SIBs) further benefit from the use of inexpensive aluminum current collectors, enabled by the non‑alloying nature of K/Na with Al, which reduces over‑discharge risks and allows safe handling at 0 V [1]. These two systems also favor transition metals with lower carbon footprint, such as Fe and Mn, over scarcer and geopolitically constrained Co, Ni, and Li [1]. In addition, the weaker Lewis acidity of K^+^ and Na^+^ compared to Li^+^ leads to reduced desolvation energy and faster ionic transport, facilitating high‑rate operation [2, 3, 4]. Similar to LIBs, anode materials for PIBs and SIBs can be broadly classified into four categories: intercalation, alloying, conversion, and conversion–alloying types. Extensive studies in the LIBs field have already established a systematic understanding of these anode chemistries, and such knowledge provides valuable insights into their potassium and sodium storage behaviors. Leveraging this mature research foundation can significantly accelerate the development of corresponding K/Na^+^ anodes, highlighting their considerable potential for future advancement [5, 6, 7, 8, 9]. As a result, research into post‑Li batteries has expanded rapidly since 2010, spurring exploration of new electrode materials and electrolytes [10].

**FIGURE 1:**
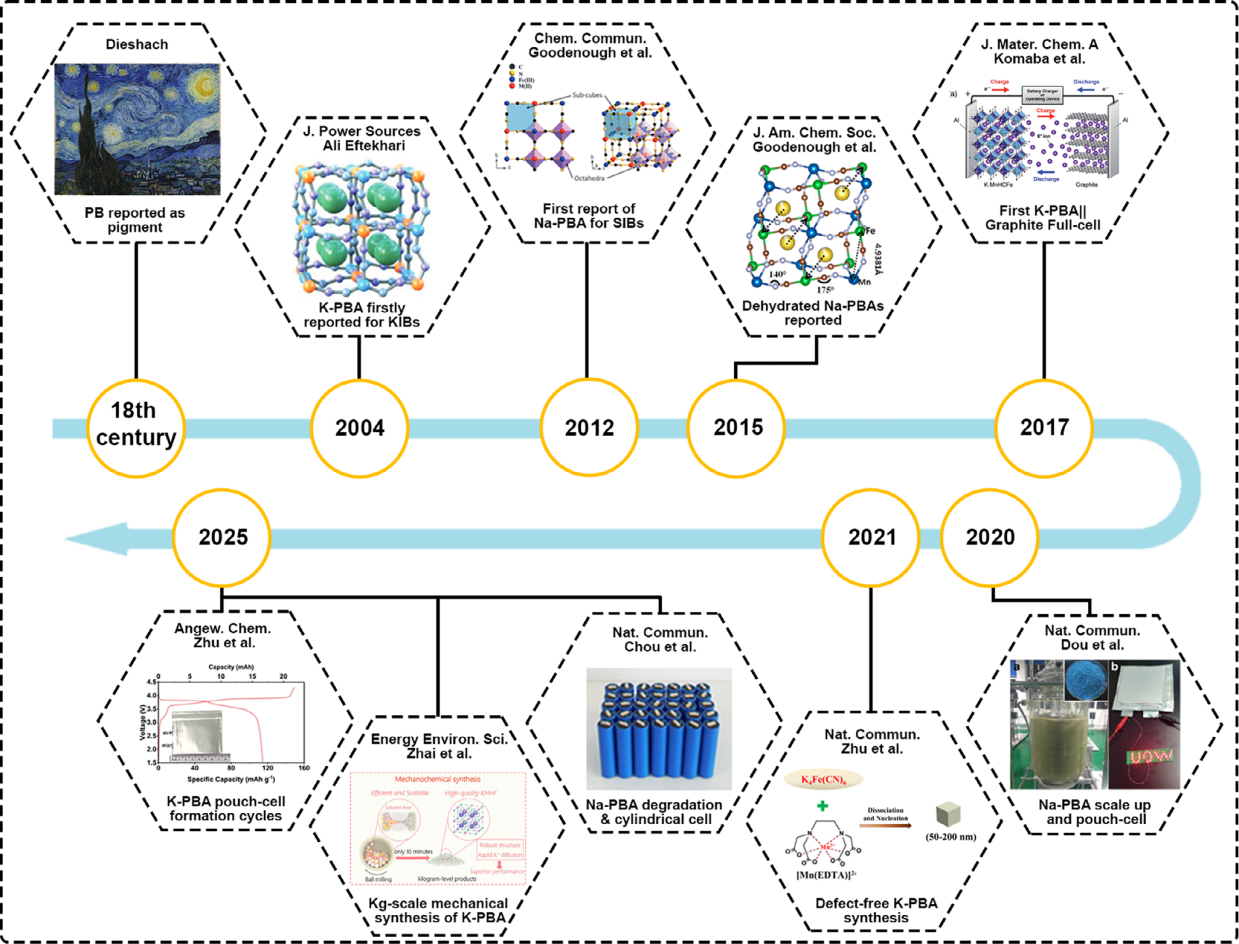
The development history of the PB and PBAs materials as cathode for PIBs and SIBs battery systems.

Despite these advantages, identifying suitable K/Na cathode materials remains challenging. Larger alkali‑ion size introduces greater lattice distortion during cycling, which can trigger structural degradation and limit long‑term stability [11, 12]. Existing cathode families, including polyanionic compounds, layered oxides, organics, and Prussian blue analogues (PBAs)—each present their own trade‑offs. Polyanionic materials offer high voltage but low capacity (<100 mAh g^−1^) due to their heavy polyanion groups, along with risks of electrolyte oxidation and Al corrosion [13, 14]. Layered oxides can deliver high theoretical capacity but often suffer from low initial alkali content, air sensitivity, inclined voltage profiles, and irreversible phase changes [12, 15]. Organic cathodes are sustainable but limited by low operating voltage, large volume change, and electrolyte dissolution issues. PBAs, first known as pigments, have emerged as a standout option for PIBs and SIBs due to their simple aqueous synthesis, abundant precursors, scalability, non‑toxicity, and balanced structural and electrochemical properties [16]. These advantages have increasingly shifted research focus toward PBAs as promising cathode materials for practical non‑aqueous batteries (Figure 1) [11, 13, 17, 18, 19, 20, 21, 22].

**FIGURE 2:**
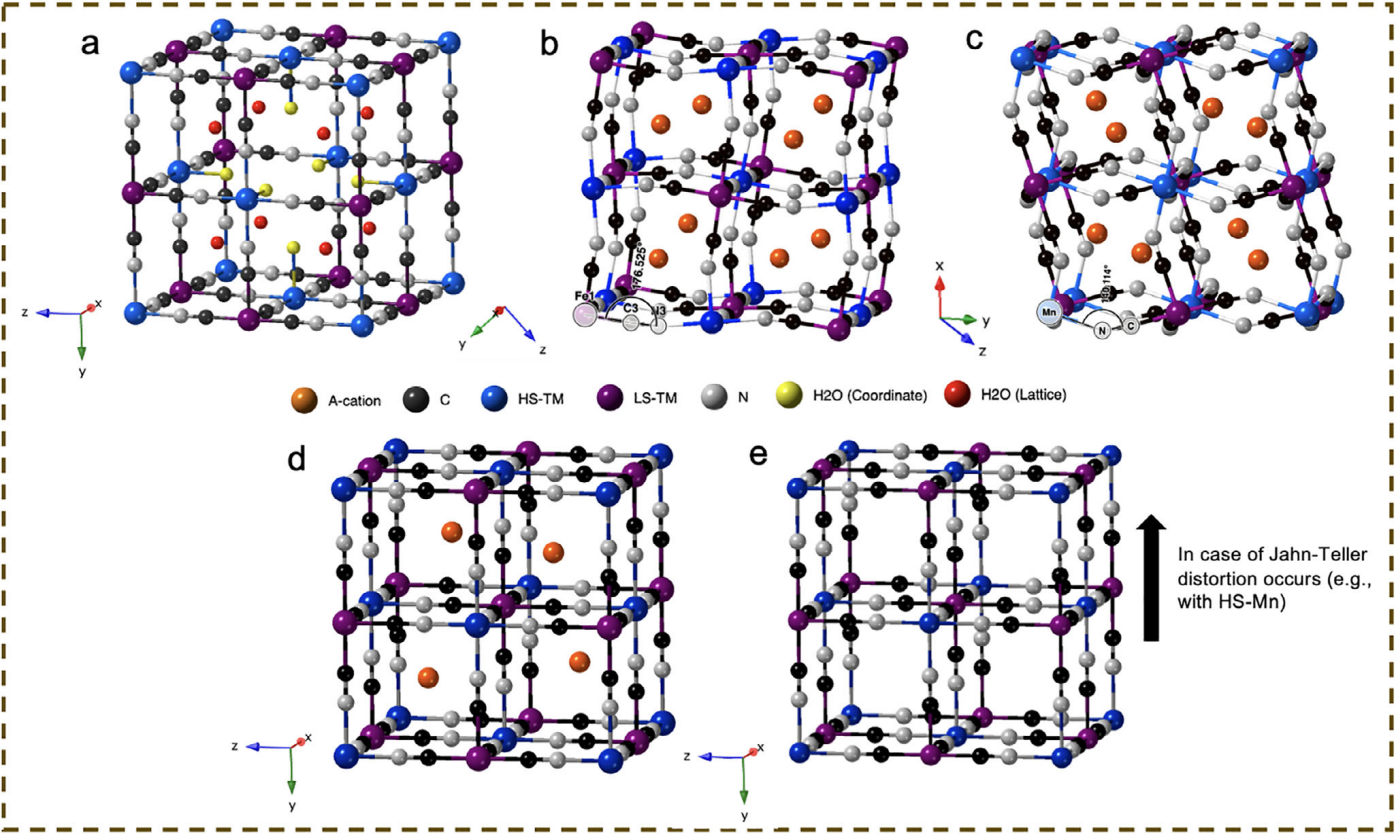
Schematic illustration of structures of (a) classic cubic PB; (b) monoclinic; (c) rhombohedral; and cubic PBAs with (d) one mole and (e) without alkaline cation present. Black, grey, blue, purple, and orange atoms represent C and N of cyanide groups, HS‐TM, LS‐TM, and alkaline cation, respectively. Water molecules are represented in red and yellow to represent lattice and coordination water, respectively.

The conventional Prussian Blue (PB) can be expressed as Fe_4_[Fe(CN)_6_]_3_·14H_2_O, the parent compound of the hexacyanoferrate (HCF) family, adopts a cubic face‑centered *Fm‑3m* structure analogous to perovskites, in which cyanide ligands bridge high‑spin (HS) Fe and low‑spin (LS) Fe centers (Figure 2a). Vacancies arising from missing [Fe(CN)_6_] units enable the accommodation of 14 water molecules—six as coordinated water bound to unsaturated HS‑Fe^3+^ and eight as lattice water within the structural voids.

In energy‑storage applications, the chemical formula of PBAs can be generally expressed as A_x_M_1_[M_2_(CN)_6_]_1−y_·zH_2_O (0 ≤ x ≤ 2, 0 ≤ y ≤ 1), where A is typically an alkaline cation and M_1_/M_2_ are transition metals (TMs) coordinated to the cyanide ligand through N and C, corresponding to HS and LS configurations. The parameter y denotes the fraction of [M_2_(CN)_6_] vacancies, which should be minimized to avoid loss of electrochemically active sites. Depending on the choice of TMs, PBAs can be grouped into single‑electron‑transfer (e.g., M_1_: Zn/Ni; M_2_: Fe/Mn/Co) or double‑electron‑transfer systems (e.g., Fe/Co/Mn combinations). The latter reach theoretical capacities of 150–170 mAh g^−1^, whereas the former offer enhanced cycling stability due to reduced structural distortion and higher conductivity [23, 24]. Owing to their robust open framework, PBAs have been explored across numerous metal‑ion chemistries (Li^+^, Na^+^, K^+^, Mg^2+^, Ca^2+^, Zn^2+^, and Al^3+^) [25, 26, 27]. Their most promising performance, however, has mostly been demonstrated in PIBs and SIBs due to their favorable compatibility and reversible alkali‑ion storage in recent years. The latest performance characteristics of some representative works of Na/K‐PBA materials are listed in Tables 1 and 2. Structural flexibility further allows K‑PBAs to crystallize predominantly in the monoclinic phase, whereas Na‑PBAs can adopt monoclinic, rhombohedral, or cubic structures depending on defect level, alkali‑ion ratio, and crystal‑water content [28, 29, 30, 31, 32, 33].

**TABLE 1:** Summary of representative PBA cathode materials in non‐aqueous PIBs focusing on chemical formula and electrochemical performance.

Chemical formula	Particle size (nm) and phase	Voltage range (V)	Initial Coulombic efficiency	Cycling performance (mAh g^−1^@mA g^−1^@retention@cycles)	Rate performance (mAh g^−1^@mA g^−1^)	Ref.
KFe[Fe(CN)_6_]	N/A	2.5–4.2	N/A	79@8@88%@500	N/A	[34]
K_1.75_Mn[Fe(CN)_6_]_0.93_·0.16H_2_O	Monoclinic, 20–30	2.0–4.5	71%	137@30@93%@100	108@1000	[32]
K_0.22_Fe[Fe(CN)_6_]_0.805_·4.01H_2_O	Cubic, 20–30	2.0–4.0	44%	73.2@50@86.5%@150	72@50	[35]
K_1.69_Fe[Fe(CN)_6_]_0.9_·0.4H_2_O	Monoclinic, ∼20	2.0–4.5	89%	140@10@60%@300	N/A	[36]
K_0.61_Fe[Fe(CN)_6_]_0.91_·0.32H_2_O	Cubic, 100	2.0–4.5	N/A	124@10@69%@500	84@500	[37]
K_1.61_Fe[Fe(CN)_6_]_0.88_·0.43H_2_O	Monoclinic, 20–60	2.0–4.0	104%	77@25@61.3%@5000	49.4@1000	[38]
K_1.94_Mn[Fe(CN)_6_]_0.994_·0.08H_2_O	Monoclinic, 50–200	2.7–4.4	81.34%	154.7@15@80%@7800	74@500	[33]
K_1.89_Mn[Fe(CN)_6_]_0.92_·0.75H_2_O	Monoclinic, 40	2.5–4.6	N/A	142.4@31.2@66%@100	N/A	[39]
K_1.66_Mn(HCF)_0.88_·0.51H_2_O@PPy	Monoclinic, 800	2.0–4.2	N/A	135@100@85%@200	92.7@1000	[40]
K_1.98_Mn[Fe(CN)_6_]_0.98_·0.24H_2_O	Monoclinic, 30–40	2.0–4.4	N/A	149@30@91.4%@500	78@7500	[41]
K_1.94_Mn[Fe(CN)_6_]_0.994_·0.08H_2_O	Monoclinic, 50–200	2.7–4.4	84.05%	132.3@15.5@> 90%@20	N/A	[42]

**TABLE 2:** Summary of representative PBA cathode materials in non‐aqueous SIBs focusing on chemical formula and electrochemical performance.

Chemical formula	Particle size (nm) and phase	Voltage range (V)	Initial Coulombic efficiency	Cycling performance (mAh g^−1^@mA g^−1^@retention@cycles)	Rate performance (mAh g^−1^@mA g^−1^)	Ref.
KMn[Fe(CN)_6_]	Hexagonal, N/A	2.0–4.0	N/A	100@10@∼100%@30	N/A	[43]
Na_0.61_Fe[Fe(CN)_6_]_0.94_·2.79H_2_O	Cubic, 300–600	2.7–4.0	N/A	170@25@∼100%@160	70@600	[44]
Na_1.92_Fe[Fe(CN)_6_]	Rhombohedral, >5 µm	2.0–4.2	>90%	160@10@80%@750	145@1500	[28]
Na_1.89_Mn[Fe(CN)_6_]_0.97_·1.87H_2_O	Rhombohedral, 200–400	2.8–4.0	N/A	150@15@75%@500	121@3000	[45]
Na_1.73_Fe[Fe(CN)_6_]·3.8H_2_O	Rhombohedral, 1.5 µm	2.0–4.0	97.4%	116@10@71%@500	70@2000	[46]
Na_1.85_Co[Fe(CN)_6_]_0.99_·1.9H_2_O	Rhombohedral, ∼600	2.0–4.1	98%	150@10@90%@200	60@500	[47]
^Na1.97Fe[Fe(CN)6]0.986^	^Monoclinic, ∼5 µm^	^2.0–4.2^	^N/A^	^156.3@17@80.6%@100^	^112@1700^	[48]
^Na1.89Mn[Fe(CN)6]0.98·0.16H2O^	^Rhombohedral, 180–450^	^2.0–4.0^	^N/A^	^161@20@92.8%@100^	^90@1000^	[49]
^Na2Fe[Fe(CN)6]^	^Rhombohedral, ∼3 µm^	^2.0–4.0^	^N/A^	^174@170@77%@50^	^115@2550^	[50]
^Na1.6Mn0.75[Fe(CN)6]·1.57H2O^	^Monoclinic, 300–400^	^2.0–4.2^	^96.9%^	^137@25@72.3%@2700^	^N/A^	[51]
^Na1.9Mn[Fe(CN)6]0.93·2.34H2O^	^Cubic, 2–4 µm^	^2.0–4.2^	^96%^	^115@25@87%@100^	^78.9@500^	[52]
^Na1.11Ni[Fe(CN)6]·0.71H2O^	^Monoclinic, 300^	^2.0–4.0^	^>90%^	^90@100@83.2%@5000^	^70.9@4000@^	[53]

^A)^ The electrochemical behavior of PBAs arises from the coupling between their framework and ion insertion/extraction. Using the ideal A_2_M_1_M_2_(CN)_6_ (x = 2, y = 0, z = 0) as an example, the redox mechanism proceeds as:.

(1) $$\begin{array}{c} A_{2}{M_{1}}^{2+}{M_{2}}^{2+}{\left(CN\right)}_{6}\to \;A{M_{1}}^{3+}{M_{2}}^{2+}{\left(CN\right)}_{6}+A^{+}+e^{-}\\ \left(\mathrm{first}\;\mathrm{charge};\;\mathrm{lower}\;\mathrm{potential}\right)\; \end{array}$$

(2) $$\begin{array}{c} A{M_{1}}^{3+}{M_{2}}^{2+}{\left(CN\right)}_{6}\to \;{M_{1}}^{3+}{M_{2}}^{3+}{\left(CN\right)}_{6}+A^{+}+e^{-}\\ \left(\mathrm{second}\;\mathrm{charge};\;\mathrm{higher}\;\mathrm{potential}\right)\; \end{array}$$

During the first charge, one alkali cation is removed, accompanied by oxidation of HS‑M_1_^2+^. A second cation is extracted at a higher potential with oxidation of LS‑M_2_^2+^, leading to a cation‑free lattice. Discharge reverses these processes through stepwise reduction of LS‑M_2_^3+^ and HS‑M_1_^3+^ with insertion of up to 2 moles of alkali‐cations.

Na‐PBAs, particularly NaFeHCFs and NaMnHCFs, exhibit complex structural evolution, as the Na content (x) commonly varies between 1 and 2 depending on synthesis conditions and dehydration state. There are three main competing interactions that need to be carefully considered to analyze the structure evolution of PBAs, which are Coulombic attraction, orbital overlap, and Pauli repulsion, where the first one leads to unit‐cell contraction and the latter two tend to alleviate the volume reduction. It is generally accepted that a low initial Na content (x) combined with a high water content (z) stabilizes the cubic structure of NaFeHCFs [54, 55], which is often considered the reference framework. Theoretical calculations suggest that in Na‐poor PBAs, Na‐ions preferentially occupy the Wyckoff 8c (body‐centered) site, while additional Na^+^ progressively fills the 24d (face‐centered) site upon insertion [56]. Increasing Na content induces a continuous 6.8% unit‐cell expansion and structural distortion into a monoclinic (*P2_1_/n*) phase (Figure 2b), until reaching the upper limit of approximately x ≈ 1.6–2.0 [57]. Upon removal of interstitial water from NaFeHCF, the framework transforms into a rhombohedral (*R‐3*) phase with more pronounced distortion (Figure 2c) [58]. The absence of crystal water—responsible for strong Pauli repulsion within the framework leads to an additional 18% contraction of the unit cell relative to the monoclinic structure. NaMnHCF exhibits a similar phase dependence on varying x and z values [45]. However, due to the larger ionic radius of Mn^2+^ (0.83 Å) compared with Fe^2+^ (0.78 Å), the Mn–N bond lengths are longer, resulting in a more pronounced unit‐cell contraction during hydration, as supported by theoretical calculations [59]. During electrochemical cycling, hydrated monoclinic and cubic Na‐PBAs first converge into a cubic Na‐poor phase upon charging, and subsequently transform into Na‐free cubic or tetragonal phases (Figure 2d–e) [46]. Notably, Goodenough's group reported that anhydrous rhombohedral Na‐PBAs follow a distinct pathway, yielding a mixture of rhombohedral and cubic Na‐poor phases during initial charging, underscoring the critical role of crystal water [28, 45].

In contrast, the introduction of larger K^+^ results in dominant Coulombic interactions that readily stabilize a more distorted monoclinic structure, even at a relatively low K content compared with an equivalent Na level and irrespective of water content, as reported in recent studies [32, 36, 39, 60, 61, 62, 63, 64]. Owing to the similar formulas of KMnHCF and KFeHCF, the unit‐cell parameter differences arising from the slight variation in ionic radii become negligible. K‐PBAs typically evolve from an initial monoclinic phase to a K‐poor cubic phase, and ultimately to a K‐free cubic or tetragonal phase when Jahn–Teller distortion occurs [32, 33].

**FIGURE 3:**
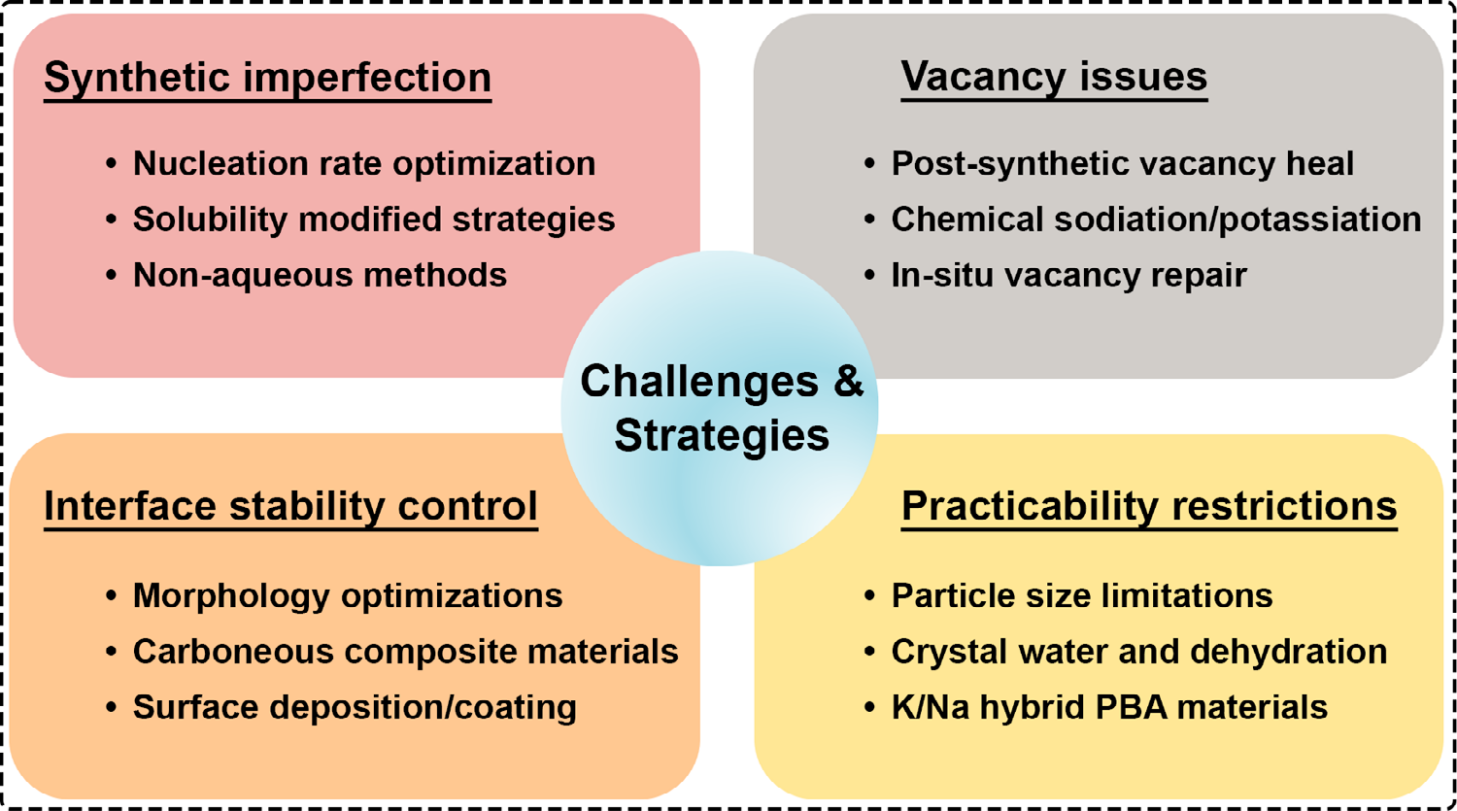
Main challenges of development of PBA materials toward a practical cathode for PIBs/SIBs.

Despite the aforementioned advantages, PBA materials still face several challenges that hinder their advancement toward practical cathode applications, as summarized in Figure 3. The primary obstacles to achieving high‐performance cathodes include intrinsic synthetic imperfections arising from conventional liquid‐phase precipitation methods, the persistent issue of TM dissolution, the resulting interfacial instability, and several practical concerns, such as crystal water retention and particle size limitations.

**FIGURE 4:**
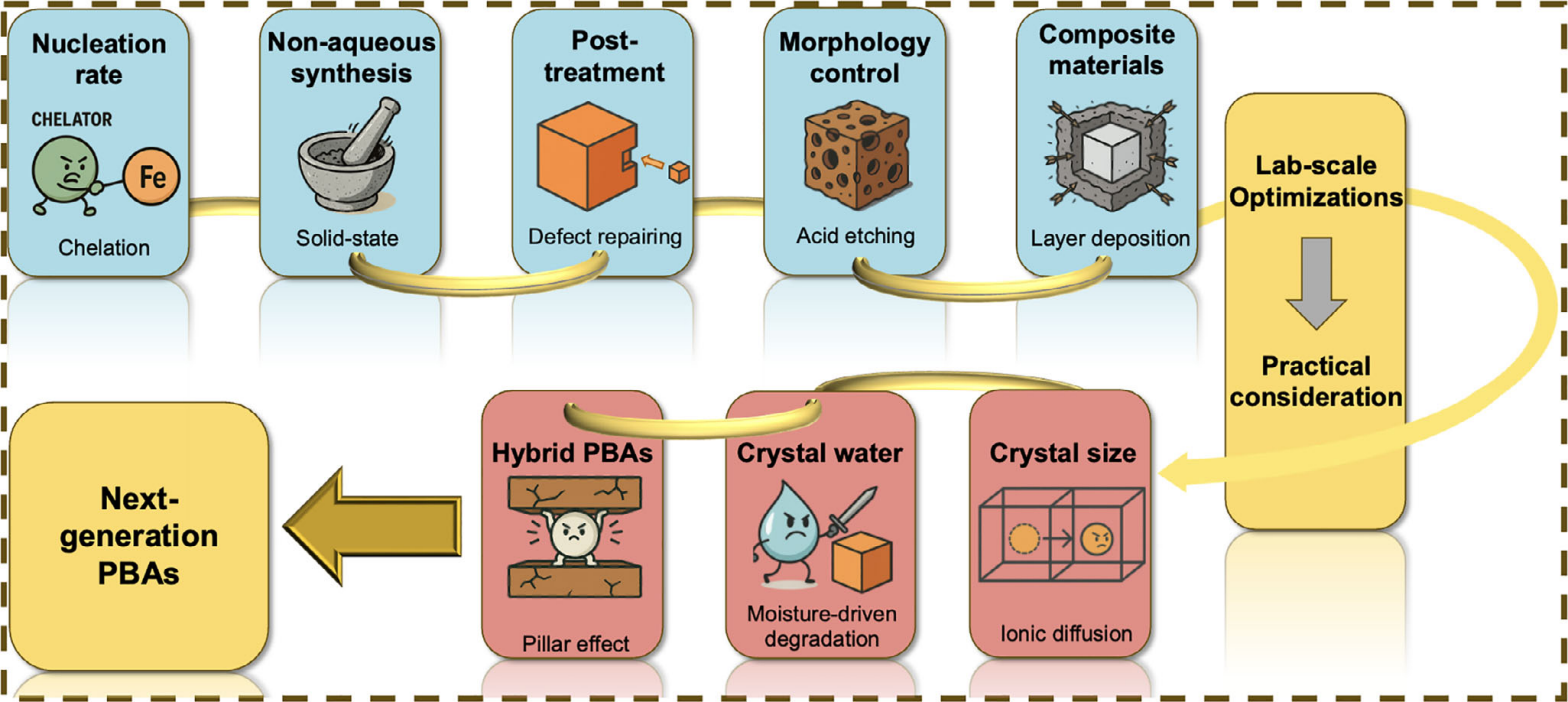
Schematics of PBAs’ transition landscape from lab‐scale modification strategies toward practical factor considerations.

Recent reviews have discussed PBA structures, diffusion mechanisms, and broad applications [65, 66, 67, 68, 69, 70]. However, key aspects relevant to practical PIB/SIB deployment, including particle‑size limitations and the role of crystal water, remain underexplored. This review, therefore, centers on synthetic strategies for producing alkaline‑rich, low‑vacancy, highly crystalline PBAs, followed by post‑synthesis modifications, surface engineering, and compositing approaches, with emphasis on their scalability constraints. We then evaluate parameters essential for practical implementation, including particle size, dehydration behavior, and the application of K‑PBAs in hybrid SIB systems (Figure 4). Considering the intertwined requirements for high‑rate capability, high operational voltage, and long cycling life, optimization strategies must be assessed holistically. Finally, we outline future perspectives at the material and full‑cell levels to bridge laboratory advances with practical PIB and SIB development.

## 2 Synthetic Optimizations Toward High‐Quality PBAs

Synthesis plays a decisive role in determining the performance and energy density of PBA cathodes. Among various preparation methods, co‑precipitation is widely viewed as the most scalable route due to its simplicity, predictability, and compatibility with processes used for LIB cathode production. In contrast, self‑precipitation and hydrothermal/solvothermal methods risk generating toxic decomposition gases such as HCN or (CN)_2_. Despite its practicality, conventional aqueous co‑precipitation often triggers rapid nucleation, producing irregular particles smaller than 50 nm with excessive Fe(CN)_6_ vacancies due to the low solubility of these complexes in water (K_sp_ ∼10^−15^) [68, 71]. Highly defective PBAs compromise battery performance by reducing active LS‑Fe^2+^/^3+^ sites, inducing lattice distortion and structural collapse, lowering initial alkali‑cation content, hindering ion diffusion through coordinated and lattice water, and catalyzing parasitic reactions that jeopardize safety [72, 73]. Lower‑vacancy PBAs also present reduced thermal‑runaway risk by limiting the release of HCN/(CN)_2_ during decomposition [74]. Since vacancy concentration correlates positively with nucleation rate, this section summarizes strategies to reduce PBA reaction kinetics in aqueous and other non‐aqueous media, including chelator selection and concentration, pH control, non‑aqueous/water‑free environments, and solid‑state synthesis.

### 2.1 Slower Nucleation and Reaction Rates

**FIGURE 5:**
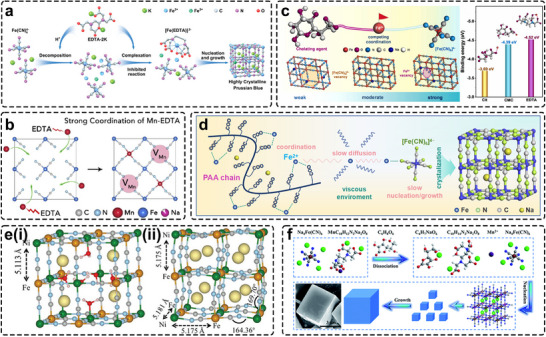
(a) Schematic illustration of coprecipitation synthesis of KFeHCF‐E with EDTA chelating agent. Reproduced with permission [38]. Copyright 2023, Wiley‐VCH. (b) The formation mechanism of Mn vacancies in NMF. Reproduced with permission [51]. Copyright 2020, Cell Press. (c) The role of competing coordination agents in the synthesis process of PBAs (left) and the calculation of the binding energies between the iron ion and different coordination agents (right). Reproduced with permission [79]. Copyright 2022, Elsevier. (d) Schematic process of HC‐PB crystallization with PAA as chelating agent [80]. Reproduced with permission. Copyright 2024, Elsevier. (e) Local structures of a) typical cubic phase NiHCF‐1 with vacancies and (b) monoclinic phase NiHCF‐3 stemmed from Rietveld refinements. Reproduced with permission [81]. Copyright 2018, Wiley‐VCH. (f) Schematic illustration of the synthesis procedure of highly crystalline Prussian white. Reproduced with permission [82]. Copyright 2019, Royal Society of Chemistry.

In aqueous co‐precipitation, anion vacancies predominantly originate from the strong thermodynamic driving force for rapid precipitation of Fe(CN)_6_‐based frameworks. Consequently, vacancy concentration is closely tied to nucleation kinetics. A common approach is to reduce the release of HS‐TM^2+^/^3+^ ions by forming stable chelate complexes (e.g., citrate, EDTA), which reduce local supersaturation (Figure 5a). Mn–citrate (*K_stable_* = 10^3.67^) and Mn–EDTA (*K_stable_* = 10^14.3^) complexes, for example, are significantly more stable than Mn–aqua species [71, 75, 76]. Using such chelators, Peng et al. synthesized Na‑rich monoclinic PBA rather than the typical Na‑poor cubic phase, achieving a capacity of 152 mAh g^−1^ compared with 120 mAh g^−1^ without chelation [48]. Thermogravimetric analysis (TGA) and Rietveld refinement confirmed reduced vacancy and water content. Xu et al. employed in‑situ FT‑IR to show that oxalate effectively suppresses rapid Fe─CN─Ni bond formation, further validating the slowed‑nucleation mechanism [77]. Beyond citrate, other chelators such as pyrophosphate (P_2_O_7_^4−^), EDTA, and CyDTA operate on the same principle [38, 77, 78]. Chelation strength can be tuned to tailor PBA properties. Shang et al. discovered that strong chelators could generate unconventional HS‐Mn vacancies by restricting Mn release, which alleviates Jahn–Teller distortion and enhances lattice stability (Figure 5b) [51]. By adjusting EDTA concentration, they produced PBAs with controlled Mn‑vacancy levels, achieving 72% capacity retention after 2000 cycles, compared with only 45% after 500 cycles for citrate‑derived samples. Furthermore, Jiang et al. proposed a balanced‑coordination strategy using sodium carboxymethylcellulose (CMC), which possesses intermediate binding strength between citrate and EDTA (DFT‑calculated, Figure 5c) [79]. The resulting low‑defect PB exhibited excellent Na‑ion kinetics, delivering 78.5% capacity retention at 100 C and a lifespan of 3000 cycles. Ion‑diffusion control provides an additional means to slow nucleation. Ma et al. identified sodium polyacrylate (PAA) as an effective additive due to its polymeric backbone and strong coordination ability (Figure 5d) [80]. Compared with citrate, PAA forms a viscous medium that restricts Fe^2+^ and Fe(CN)_6_^4−^ diffusion, significantly decelerating nucleation and promoting high crystallinity in the final PBAs.

Beyond selecting appropriate chelators, their concentration plays a decisive role in improving PBA quality. Increasing chelator concentration gradually shifts PBAs from a Na‐poor cubic structure (Figure 5e (i)) to a Na‐rich monoclinic phase (Figure 5e(ii)), where the C─N─Ni bond angle decreases from 180° to 169°, and the lattice undergoes only ∼3% expansion during Na insertion [81]. DFT calculations indicate that this distortion narrows the electronic bandgap, enhances Fe redox activity, and improves electronic conductivity, serving as a useful indicator of initial Na content and defect concentration. Na‐rich monoclinic PBAs also demonstrate superior compatibility and safety for practical operation, exhibiting thermal stability beyond 300°C compared with ∼250°C for cubic analogues [83]. The speciation and competition of ligands are strongly pH‐dependent. Increasing pH deprotonates carboxylate groups, enhancing the binding affinity of citrate, EDTA, and related ligands toward metal cations and further slowing nucleation. Using sodium acetate and acetic acid to regulate acidity, Neale et al. reported that PBAs synthesized at higher pH crystallized into larger cubic particles with fewer vacancies [76]. Similarly, Peng et al. employed ascorbic acid as a pH regulator, and the solution containing MnNa_2_‐EDTA and Na_4_Fe(CN)_6_ was titrated dropwise (Figure 5f) to gradually weaken the strong binding of EDTA and enable controlled nucleation [82]. The steric hindrance of EDTA^4−^ further suppressed aggregation by adsorbing onto the nuclei surface. This strategy produced a well‐crystallized H‐PBM with higher tap density, substantially improved long−term cycling stability of 82% after 500 cycles, and a stable capacity of 128 mAh g^−1^, along with reversible monoclinic‐to‐cubic phase transition, compared to the uncontrolled co‐precipitated L‐PBM. As discussed earlier, weakly chelating citrate salt solutions are typically basic and must be used in large quantities to reduce LS‐TM vacancies, whereas strongly chelating EDTA salt solutions are slightly acidic and tend to hinder the release of TM ions, leading to HS‐TM vacancies. Regulation of the reaction pH, therefore, provides an effective means to reduce chelator consumption and the associated processing costs. By tuning the ratio of potassium citrate (citrate‐3K) to EDTA‐2K to 3:1, Yue et al. optimized the coordination environment and precisely controlled the nucleation kinetics [84]. This strategy markedly increased the content of LS‐Fe in PBAs while reducing lattice defects, thereby enhancing structural integrity and electrochemical stability. The optimized KCA75 electrode delivers a high initial capacity of 128 mAh g^−1^ and exhibits excellent cycling durability, retaining 94.8% of its capacity after 1000 cycles at 5 C.

### 2.2 Non‐Aqueous, Water‐Less and Solubility‐Modified Synthesis

Conventionally, the rapid nucleation and growth of PBAs in aqueous media generate substantial crystal defects. This increases the likelihood of internal water incorporation because of the high solubility of reagent salts. Although the use of chelators, as outlined in the previous section, is an effective approach to producing PBAs with fewer defects and reduced crystal water content, the cost associated with both chelator consumption and subsequent recycling remains significant. Three strategies have therefore been adopted to reduce reliance on chelators and mitigate the drawbacks of aqueous synthesis: solubility‐modified routes, non‐aqueous or water‐lean liquid‐phase synthesis, and solid‐state methods.

**FIGURE 6:**
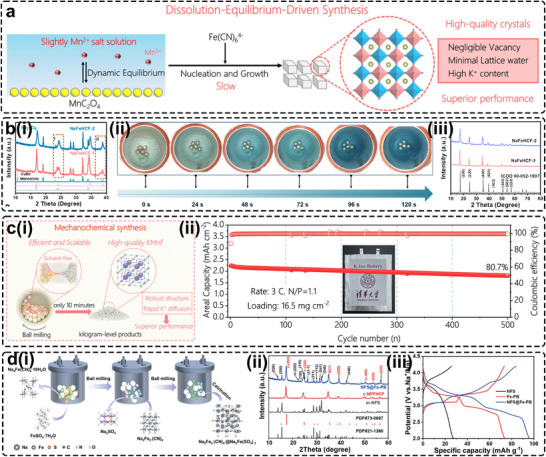
(a) Schematic diagrams of the dissolution–equilibrium driven synthesis strategy. Reproduced with permission [85]. Copyright 2025, American Chemical Society. (b) (i) The XRD patterns of NaFeHCF‐1 and NaFeHCF‐2 precursors before washing; (ii) The oxidation process of freshly made PBA powder over time; (iii) The XRD patterns of NaFeHCF‐1 and NaFeHCF‐2 precursors after washing and drying. Reproduced with permission [87]. Copyright 2022, Elsevier. (c) (i) Schematic diagram of the process and technical characteristics of mechanochemical synthesis; (ii) Cycling stability of the KMnHCF‐MC//PTCDI pouch cell with high area capacity at a constant charge–discharge rate of 3 C (1 C = 150 mAh g^−1^). Reproduced with permission [41]. Copyright 2025, Royal Society of Chemistry. (d) (i) Schematic illustration of the synthesis of NFS@Fe‐PB sample based on mechanochemical ball milling strategy; (ii) XRD patterns of NFS@Fe‐PB and its components; (iii) Galvanostatic charge/discharge profiles measured at 10 mA g^−1^. Reproduced with permission [88]. Copyright 2025, Wiley‐VCH.

A recent example is a dissolution–equilibrium‐driven synthesis reported by Wang et al., in which high‐quality KMnHCF was produced by employing the sparingly soluble Mn source MnC_2_O_4_ instead of Mn(Ac)_2_ in aqueous solution (Figure 6a) [85]. Dynamic equilibrium between dissolved Mn^2+^ and solid MnC_2_O_4_ moderated crystallization kinetics without the need for chelating agents, thereby eliminating waste complexants requiring disposal. The resulting H‐MnPBA exhibited negligible vacancy content and suppressed Jahn–Teller distortion, delivering stable cycling retention of 75.39% after 1000 cycles in half‐cells and an energy density of 306 Wh kg^−1^ in full cells paired with a graphite anode [85]. Similarly, precipitation rates can be suppressed by exploiting the lower solubility of reactants in organic solvents. Using ethylene glycol (EG) as the reaction medium achieved this effect, although the limited solubility of Na salts in organic media led to low initial Na content in both PBAs [86]. Future work should prioritize identifying suitable organic solvents by evaluating their solvation strength and the solubilities of all relevant precursors to optimize reaction environments and improve PBA quality.

Opposite to liquid‐phase synthesis, solid‐state methods such as ball‐milling are considered efficient and economical for producing PBAs because of their high space‐utilization rate. Owing to the minimal use of water as solvent or milling medium, the risk of internal water incorporation is greatly reduced [89]. Using a “water‐in‐salt” nano‐reactor, Peng et al. achieved a PBA with markedly improved air stability and all‐climate electrochemical performance compared with the co‐precipitated sample, while reducing water consumption by almost fiftyfold [89]. The resulting material exhibited small particle size and high crystallinity, retaining 75% of its original capacity at 500 mA g^−1^ even at −10°C. Zhang et al. showed that Ar protection during ball milling was critical for suppressing HS‐Fe oxidation and producing a Na‐richer PBA (NaFeHCF‐1) prior to washing, relative to the sample synthesized under air (NaFeHCF‐2) (Figure 6b(i)) [87]. However, by‐product removal and sample handling remained challenging. Solvent choice also required careful consideration because freshly formed monoclinic PBAs were highly sensitive to moisture as exposure to humid air induced Na loss and transformed the monoclinic phase into the cubic structure through oxidation (Figure 6b(ii,iii)). In addition to these processing issues, He et al. found that precursor dehydration hindered target‐phase formation during mechanochemical reactions [90]. They proposed that a small amount of water present before phase formation aids structural stabilization within the PBA lattice. Considering these factors, Wang et al. developed a water‐free strategy to synthesize kilogram‐scale K_2_Mn[Fe(CN)_6_] within 10 min (Figure 6c(i)) [41]. The resulting KMF delivered an energy density of 590 Wh kg^−1^ at 0.2 C, demonstrated cycling stability over 10 000 cycles, and exhibited rate capability up to 50 C in a potassium‐metal half‐cell. A pouch cell with an areal capacity of 2.2 mAh cm^−2^ achieved 500 cycles with 80.7% capacity retention (Figure 6c(ii)). This method also enabled the synthesis of analogous PBAs by substituting Mn with Mg or Ca, broadening the practical applicability of the approach. To eliminate both by‐product influence and the washing step, Gao et al. reported a “zero‐waste” solid‐state protocol [88]. In this strategy, the unwashed ball‐milled PBA was directly calcined at low temperature (Figure 6d(i)), producing a composite material (NFS@Fe‐PB) comprising polyanionic monoclinic Na_2_Fe(SO_4_)_2_ (m‐NFS) and cubic PBA (c‐NFFHCF), as confirmed by XRD (Figure 6d(ii)). The phase fractions of PBA and m‐NFS were refined to 62 and 38 wt.%, respectively. Their synergistic interaction enabled highly reversible phase transitions, a robust structural framework, and rapid Na‐ion storage, delivering 94 mAh g^−1^, outperforming their individual components (Figure 6d(ii)). The composite also achieved stable performance from −10 to 50°C, offering a cost‐effective and scalable route for large‐scale PBA production [88].

Despite these advancements, PBAs synthesized via high‐energy mechanochemical routes generally exhibit very small particle sizes of typically 50 to several hundred nanometers, as particle growth is restricted by intense grinding forces. Although small particles support fast ion and electron transport and enhance rate performance, they require more binder to ensure sufficient cohesion in thick electrodes. Without adequate binding, electrodes are prone to cracking, which is problematic for high‐energy cells that require large electrode loadings. Furthermore, the added cost associated with Ar protection during synthesis and handling diminishes the scalability advantage of solid‐state processing, representing key drawbacks that hinder its commercialization.

## 3 Post‐Synthesis Optimizations

Although the previous sections outlined strategies for producing PBAs with minimal defects and nearly stoichiometric alkaline‐cation content, conventional single‐step synthesis may still fall short of delivering the desired material quality. Post‐synthesis optimization, therefore, serves as a valuable final step to further upgrade the as‐prepared PBAs.

**FIGURE 7:**
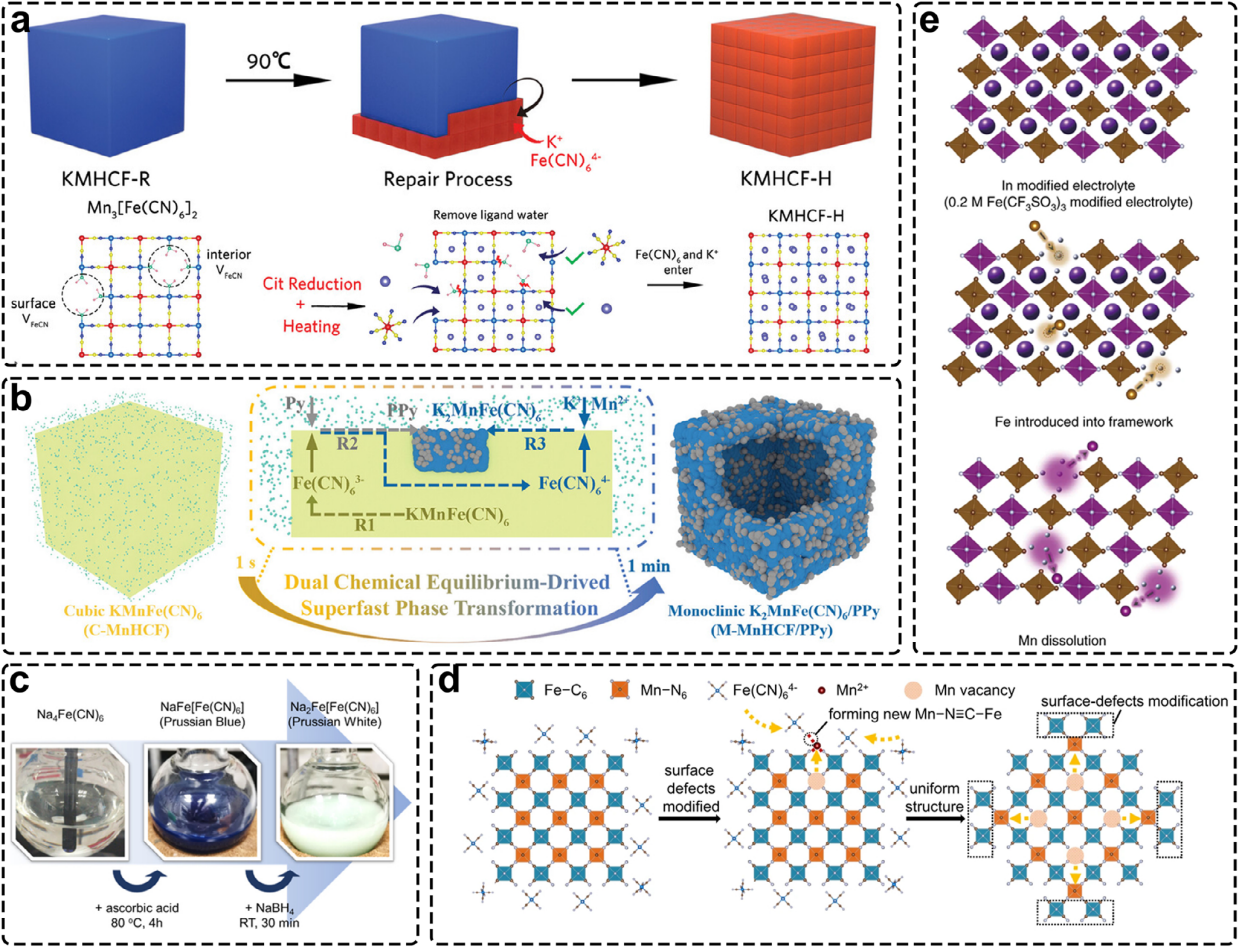
(a) Schematic diagram of structural evolution from the KMHCF‐R cube precursor to the KMHCF‐H assembled cube. Reproduced with permission [93]. Copyright 2024, Wiley‐VCH. (b) Schematic illustration of the dual chemical equilibrium−driven phase transformation process. Reproduced with permission [40]. Copyright 2024, Wiley‐VCH. (c) Reaction scheme for the synthesis of PB, followed by its conversion into PW. Reproduced with permission [50]. Copyright 2021, American Chemical Society. (d) Schematic illustration of the cation‐trapping process. Reproduced with permission [95]. Copyright 2023, Springer Nature. (e) Schematic of our surface‐modification strategy for mitigating structural instability due to Mn dissolution in the KMnHCF electrode. Reproduced with permission [73]. Copyright 2021, Springer Nature.

For instance, vacancies formed during rapid nucleation can be partially repaired through solid‐state milling of defective PBA Fe^3+^[Fe^2+^(CN)_6_]_3/4_, which contains 25% vacancies, together with Na_4_Fe(CN)_6_ as the joint Na^+^ and [Fe(CN)_6_]^4−^ source [91]. This behavior is consistent with observations by Cattermull et al. [92]. However, vacancy filling during solid‐state reaction is often kinetically limited and can terminate prematurely because the diffusion of K^+^ and/or [Fe(CN)_6_]^4−^ anions becomes blocked even after prolonged processing. Liquid‐phase repair offers a different scenario. In principle, the solution provides a medium in which ions can move freely in and out of the PBA lattice. Li et al. demonstrated that elevated temperature was essential for detaching coordinated water from Mn sites, thereby exposing them to the repair reagent and enabling vacancy healing in core regions [93]. Using citric acid as a mild reducing agent at high temperature (Figure 7a), [Fe(CN)_6_]^3−^ units in the Mn[Fe(CN)_6_]_2/3_ (MFC) precursor were gradually reduced to [Fe(CN)_6_]^4−^, which facilitated the incorporation of K^+^ and [Fe(CN)_6_]^4−^ into defect sites. This process lowered the defect level to only 2% in K_1.82_Mn[Fe(CN)_6_]_0.98_·0.58H_2_O (KMnHCF‐H). The repaired PBA promoted preferential electrolyte–salt decomposition, forming an intact cathode–electrolyte interface (CEI) that effectively suppressed Mn dissolution. As a result, KMnHCF‐H delivered excellent cycling stability in both half cells (95.2% retention at 0.2 A g^−1^ after 2000 cycles) and full cells (99.4% retention at 0.1 A g^−1^ after 200 cycles). Wan et al. further showed that defective PBAs could be post‐treated in a highly concentrated Na_4_Fe(CN)_6_ precursor solution [94]. The resulting material exhibited 20% fewer vacancies than the pristine sample, consistent with theoretical calculations indicating that exposed HS‐Fe sites possess the highest binding energy toward [Fe(CN)_6_]^4−^, thereby thermodynamically favoring vacancy reduction.

Upon defect remediation, the alkaline content of PBAs can be further enriched to 2 moles by introducing a reductive compound in the presence of the corresponding alkaline cation. Dai et al. employed a precipitation–dissolution and redox equilibrium‐driven phase transformation between Prussian Blue (PB) KMnFe(CN)_6_ and polypyrrole (ppy) to yield Prussian White (PW) K_2_MnFe(CN)_6_ (Figure 7b) [40]. In this process, ppy was spontaneously oxidized by HS‐Mn^3+^, accompanied by the diffusion of K^+^ ions. This “one‐stone‐two‐birds” strategy enabled the formation of a conformal ppy coating on PBA particles, simultaneously enhancing electronic conductivity and facilitating K‐ion insertion kinetics at higher potentials. Comparative studies by Guo's group revealed that N_2_ bubbling and the addition of ascorbic acid during acid self‐precipitation were essential to obtain Na‐rich monoclinic NaFe(1.63) [44, 97], which initially contained 1.63 mol Na. Similarly, stronger reductants such as NaBH_4_ and NaI were employed by Cheryldine et al. and Brant et al. to convert PB NaFeFe(CN)_6_, synthesized via self‐precipitation without atmospheric control [50, 58], into the corresponding Prussian White (Figure 7c) Na_2_FeFe(CN)_6_. This dehydrated Na‐rich PBA represents a typical reduced phase. These two‐step reduction routes offer viable alternatives to high‐temperature, high‐pressure, or oxygen‐free synthesis, thereby lowering processing cost and complexity. Furthermore, Zheng et al. recently reported a chemical pre‐sodiation approach in which a coated electrode composed of cubic Na‐poor PBA Na_0.37_Fe[Fe(CN)_6_]_0.86_ was directly treated with sodium benzophenone [98], yielding a Na‐rich hydrated Prussian Blue (H−PB) with expelled crystal water and eliminating the need for a separate drying step for both powder and electrode. However, additional costs associated with the use of reductive reagents, inert‐environment operation, and potential incompatibility with electrode components (e.g., carbon black and binders) must be carefully addressed to prevent accelerated cell degradation [99].

Although the aforementioned strategies effectively reduce initial defects and improve crystallinity and stoichiometry, the gradual dissolution of HS transition‐metal elements remains a persistent issue for high voltage cathodes such as Mn‐HCFs (where CEI needs to be more robust to protect it from electrolyte attack), especially when Jahn–Teller distortion of Mn is present. Dissolved cations can migrate and deposit on the anode surface, disturbing the stabilities of both solid–electrolyte interphase (SEI) and CEI, which leads to increased impedance or even cell failure. For example, the CEI formed on PBM (PBA@MXene) with suppressed Mn dissolution is uniform and dense [100], whereas the CEI on bare PBA is rough and significantly thickened after only 10 cycles. In the absence of MXene as a surface protector, the fragile CEI on bare PBA cannot effectively shield the electrode from electrolyte corrosion and is prone to severe cracking. Such a defective CEI layer not only fails to protect the active material from electrolyte‐induced oxidation but also introduces additional charge‐transfer impedance, thereby hindering the rapid ionic diffusion required for practical fast charging. Consistent with this interpretation, Feng et al. investigated the interfacial properties using electrochemical impedance spectroscopy (EIS) and found that the charge‐transfer resistance (R_ct_) of the cycled PBA@PBN electrode was approximately four times lower than that of bare PBA with much superior rate performance, in which severe Mn dissolution was observed [101]. These results clearly demonstrate that Mn dissolution substantially compromises interfacial robustness and, in turn, impedes rate performance and fast‐charge ability.

Liang et al. proposed that such dislocated cations can be captured by the electrolyte additive Na_4_Fe(CN)_6_, thereby mitigating or preventing metal dissolution [95]. Once Mn dissolution occurs, the released Mn^2+^ ions are trapped by the low−concentration supporting salt in a 17.6 m NaClO_4_ electrolyte and recrystallize into a new electrochemically active framework on the PBA surface (Figure 7d). This process maintains structural integrity and yields superior elemental uniformity compared with conventional doping and electrolyte−engineering approaches. In contrast to cation trapping, Ge et al. developed an in situ surface elemental substitution strategy by introducing Fe(CF_3_SO_3_)_3_ into a 21 m KCF_3_SO_3_ electrolyte (Figure 7e) to repair HS transition‐metal vacancies in the K_1.82_Mn[Fe(CN)_6_]_0.96_ cathode [96]. The substituted Fe‐containing PBA formed a protective surface layer that served as a structural shield while generating a KF‐rich CEI, which effectively suppressed Mn dissolution and lattice distortion, thereby preserving structural stability and reversible K‐storage capacity. Although these methods are currently limited by the low solubility of such salts in non‐aqueous electrolytes, similar strategies may be extended through computational screening to identify alternative additives with sufficient solubility and trapping capability. Instead of introducing trapping additives that increase manufacturing complexity and cost, Wang et al. demonstrated that Mn dissolution can be mitigated at its origin by employing tetramethylene sulfone (SL), a solvent with intrinsically lower solubility for Mn‐PBAs [102]. In this electrolyte, the formation of a dissolution–diffusion inhibition interface kinetically suppresses Mn dissolution by an order of magnitude compared with conventional solvents such as propylene carbonate (PC). This approach enables a high specific capacity of 154 mAh g^−1^ and stable cycling over more than 1000 cycles at 2 C.

Beyond electrolyte engineering, the interface between the PBA cathode and electrolyte also plays a crucial role. Leveraging the strong coordination between Mn ions and sodium alginate (SA) macromolecules, Ge et al. constructed a cross‐linked network, [Mn(SA)_n_], that firmly anchors Mn ions and shields them from electrolyte attack [103]. This strategy yields a nearly sixfold decrease in Mn dissolution. The oxygen‐containing groups (─OH and ─COOH) further facilitate charge transfer and enhance reaction kinetics, preserving structural integrity and supporting stable cycling of Mn‐SA for 1000 cycles.

## 4 Surface Modifications and Compositing PBAs

PBAs that crystallize into large cubic particles often experience sluggish kinetics due to their intrinsically low electronic conductivity and slow ionic transport, which results in long diffusion pathways with increasing depth from the surface. Achieving both small particle size and high crystallinity is inherently challenging, as stoichiometric PBAs typically form large crystals with limited surface area. To overcome these trade‐offs, researchers have developed diverse surface‐engineering and compositing strategies to enlarge the effective surface area, thereby shortening ion‐insertion pathways, while enhancing electronic conductivity through conductive‐polymer coatings or integration with carbon‐based materials. These approaches aim to construct PBAs with optimized microstructures that deliver superior rate capability and improved electrochemical performance.

### 4.1 Morphology Control

Inspired by the understanding that enhanced charge transfer and ion diffusion can be achieved by increasing specific surface area, surface‐engineered PBAs have been designed to tailor particle morphology and thereby improve the balance between energy and power densities. Surface modification strategies primarily involve morphology control through self‐assembly, templating, or surface etching, with the goal of creating porous or hollow architectures and tunable internal microstructures. Such designs facilitate rapid ionic transport, accommodate volume variation during cycling, and enhance electrolyte penetration and wetting through interconnected mesopores, collectively contributing to improved electrochemical performance [104, 105].

**FIGURE 8:**
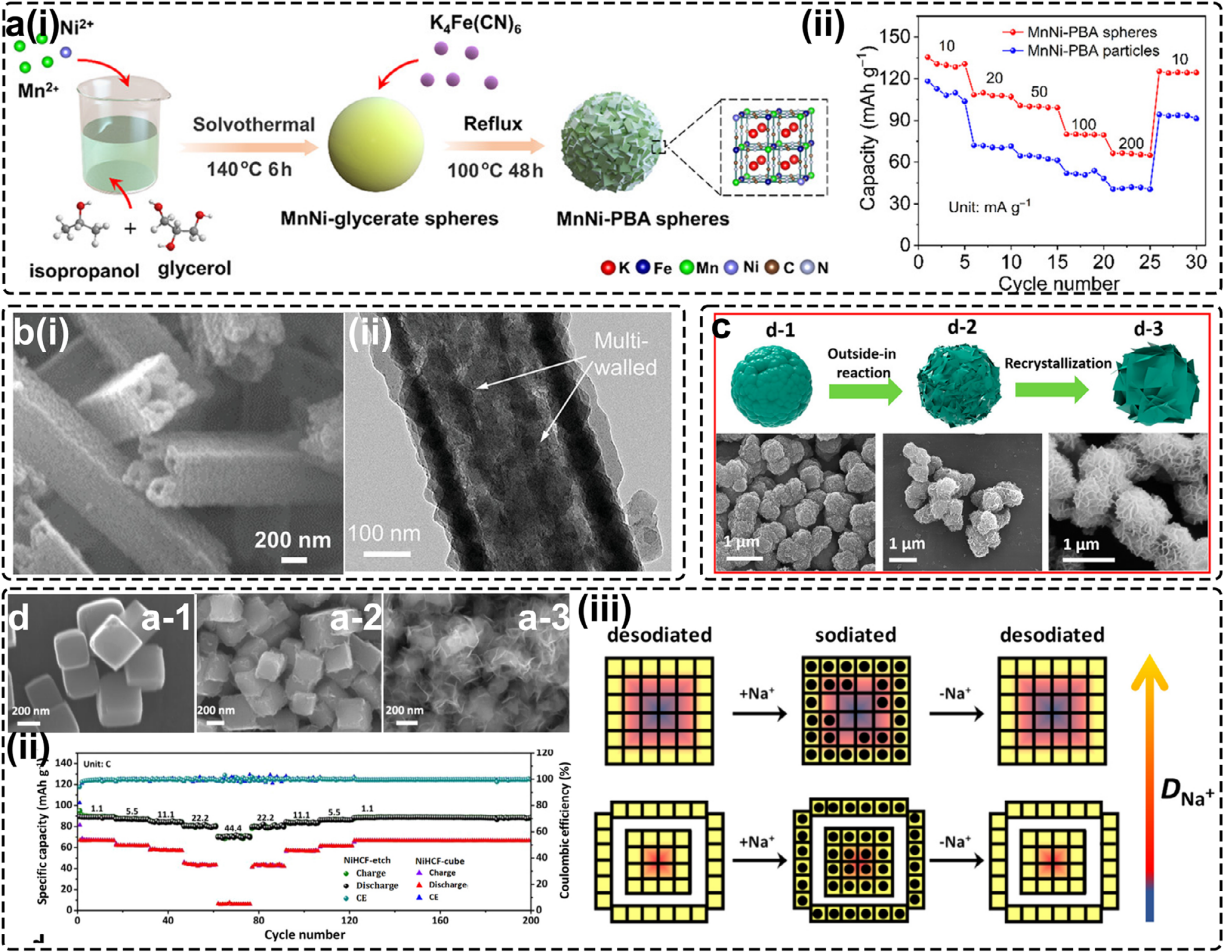
(a) (i) Schematic illustration of the formation procedure of MnNiHCF spheres; (ii) Rate capabilities of the MnNiPBA spheres and the MnNi‐PBA particles. Reproduced with permission [106]. Copyright 2022, American Chemical Society. (b) (i) Cross‐section FESEM images of PW‐HN. (ii) TEM image of PW‐HN. Reproduced with permission [107]. Copyright 2019, WILEY‐VCH. (c) Schematic diagram and the corresponding SEM images of the as‐synthesized Prussian blue materials aged for **(d‐1)** 3, **(d‐2)** 6, and **(d‐3)** 48 h. Reproduced with permission [108]. Copyright 2019, American Chemical Society. (d) field‐emission scanning electron microscope (FESEM) images of the NiHCF products after the etching time for **(a‐1)** 0 h, **(a‐2)** 0.5 h, **(a‐3)** 6 h; (ii) Rate performance of NiHCF‐cube and NiHCF‐etch; (iii) Outside‐in diffusion route of Na‐ions in NiHCF‐cube and NiHCF‐etch. Reproduced with permission [53]. Copyright 2017, American Chemical Society.

By employing MnNi‐glycerate spheres synthesized via a solvothermal route as a templating precursor, Li et al. produced a unique porous spherical MnNi‐PBA (Figure 8a(i)) [106], analogous to the NaFeHCF derived from hollow Fe^2+^O_x_ nanospheres reported by Wang's group [24]. Owing to its large specific surface area (46.16 m^2^ g^−1^), the hierarchical architecture comprising micron‐sized secondary particles assembled from nanosized subunits offers both compatibility with commercial electrode‐coating processes and enhanced electrolyte contact. Consequently, the material exhibited superior cycling stability (88.4% after 300 cycles) and rate capability (130.7/66.3 mAh g^−1^ at 10/200 mA g^−1^) compared with conventional cubic PBAs (Figure 8a(ii)). Ren et al. developed a self‐templated approach to synthesize a novel tubular PBA via an inside‐out Ostwald‐ripening mechanism, in which the metastable core dissolves and recrystallizes onto the outer surface [107]. The resulting nanotubular NaFeHCF featured micrometer‐scale lengths and widths of ∼ 400 nm, composed of ordered hollow blocks (30–50 nm) (Figure 8b(i,ii)). This hierarchical morphology enabled pseudo‐capacitive dominated Na‐storage behavior with negligible volume expansion, delivering exceptional rate capability (83 mAh g^−1^ at 50 C) and cycling stability (80% after 5000 cycles at 10 C). Mai's group further demonstrated a flower‐like PBA assembled from nanosheets (PB‐NSs) [108], synthesized via decomposition of K_3_Fe(CN)_6_ under HCl‐assisted etching followed by slow recrystallization. As shown in Figure 8c(d_1_‐d_3_), prolonged resting time promoted nanoflake extension along the basal planes, eventually forming a mesoporous PBA after 48 h with an enlarged surface area of 111.8 m^2^ g^−1^. The increased diffusion coefficient (D^GITT^) confirmed improved K‐ion diffusivity, resulting in enhanced electrochemical performance. Similar to commercial Li‐ion cathodes, the tailored morphology, particularly the formation of secondary particles, has a pronounced impact on practical properties such as tap density. Fan et al. proposed a secondary‐particle granulation strategy driven by controlled recrystallization. The resulting micrometer‐sized monoclinic hexacyanoferrate (CFHCF) [109], featuring a quasi‐spherical morphology composed of nanosized primary particles, achieved a remarkably high tap density of 0.992 g cm^−3^. This optimized architecture simultaneously enhanced ionic diffusivity and enabled excellent pouch‐cell performance.

Because PBAs are interconnected by cyanide bridges, their frameworks are inherently fragile and prone to corrosion under acidic or basic conditions that disrupt the TM‐NC‐TM linkages. Time‐dependent studies by Ren et al. revealed a defect‐induced morphological evolution from nano‐cube to nanoflower structures (Figure 8d(a_1_‐a_3_)) [53]. Upon exposure to NaOH solution for 0.5 h, the edges of NiHCF cubes were preferentially dissolved, and subsequent dissolution–recrystallization over 6 h led to the formation of nanoflower‐like structures. The resulting morphology exhibited higher capacity and faster Na‐storage kinetics, maintaining 78% of its capacity even at a high current density of 44.4 C (Figure 8d(ii)). As illustrated in Figure 8d(iii), this enhancement arises from increased accessibility of reaction sites previously confined within the particle core, as Na‐ion diffusion proceeds via an “outside‐in” pathway, thereby shortening ion‐transport distances.

A notable drawback of these porosity‐engineering approaches is that the introduction of pore defects can initially compromise cycling stability. The enlarged surface area increases the likelihood of side reactions with the electrolyte, elevating safety risks. Moreover, excessive porosity inevitably lowers the tap density of PBA electrodes, which can hinder volumetric energy density and pose challenges for commercial electrode fabrication. Therefore, the degree of porosity must be carefully optimized to balance electrochemical performance with practical process compatibility.

### 4.2 Compositing With Carbon Materials and Surface Deposition

Beyond surface‐area enlargement, compositing with secondary materials such as functional surface coatings or carbon‐based frameworks has emerged as an effective strategy to enhance the electrochemical performance of PBAs. This approach offers several key advantages: (i) integration with conductive materials or surface−deposited layers improves overall electronic conductivity and rate capability [78]; (ii) flexible composite matrices can accommodate volume expansion and alleviate structural strain during Na^+^/K^+^ (de)intercalation [110]; and (iii) the composite layer serves as a physical barrier that mitigates side reactions with electrolytes and suppresses transition‐metal dissolution [40].

**FIGURE 9:**
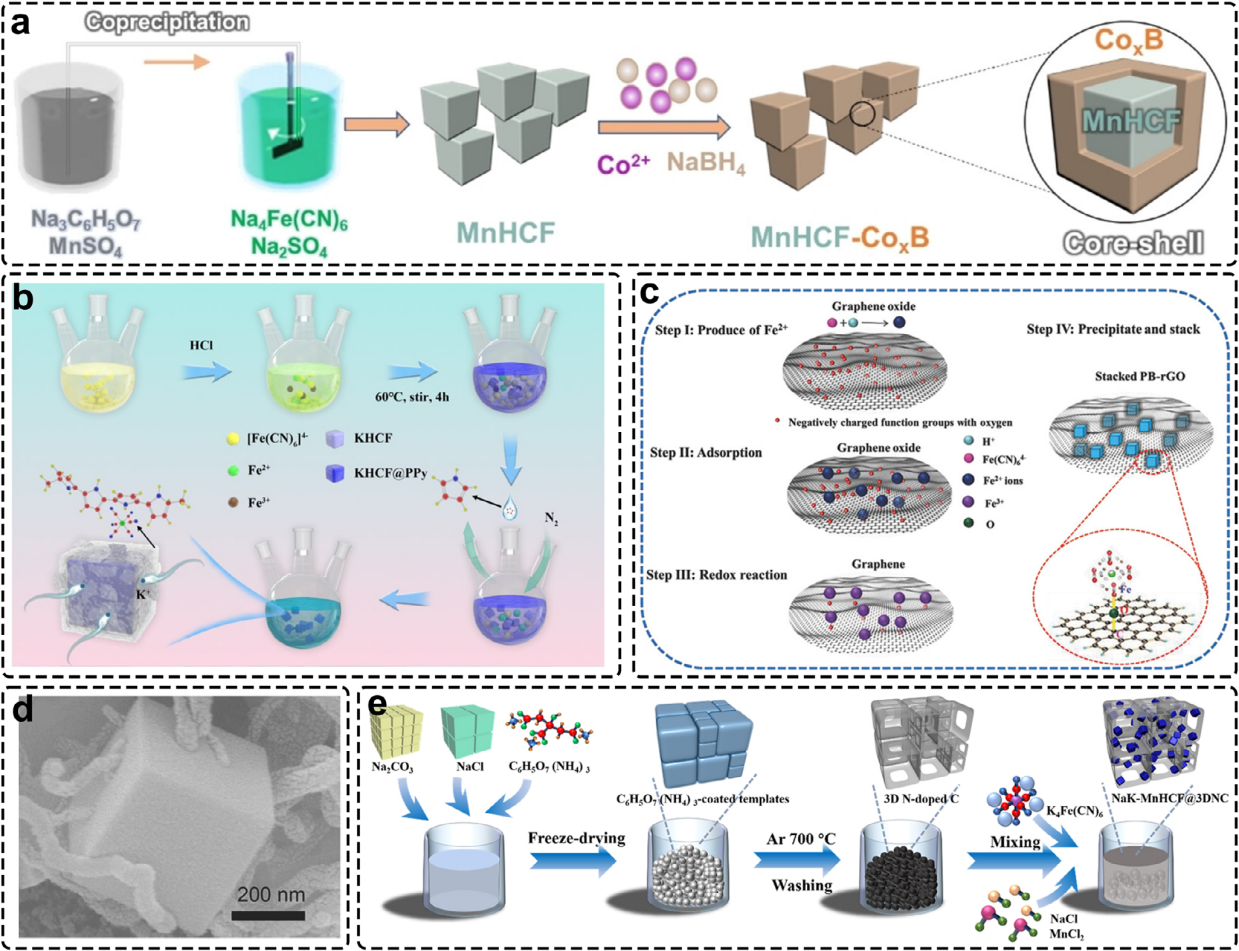
(a) Schematic illustrations of the synthesis process and architecture of the core–shell MnHCF‐CoxB cathodes. Reproduced with permission [111]. Copyright 2023, Wiley‐VCH. (b) Illustration scheme of the synthesis process with spontaneous polymerization initiated by oxidized HS‐Fe^3+^. Reproduced with permission [115]. Copyright 2019, American Chemical Society. (c) The mechanism of the synthetic process of PB‐RGO composite. Reproduced with permission [116]. Copyright 2018, Wiley‐VCH. (d) SEM images of the PB/CNT. Reproduced with permission [117]. Copyright 2016, WILEY‐VCH. (e) Schematic illustration of the synthesis procedure of the NaK‐MnHCF@3DNC compounds. Reproduced with permission [118]. Copyright 2019, Elsevier.

Given the low thermal stability of PBAs, conventional pyrolytic carbon‐coating methods (>350°C) are unsuitable, which significantly constrains coating options [74]. Applicable coating materials must therefore form at low temperatures while remaining chemically stable with cell components. Xu et al. developed an inorganic core–shell Co_x_B‐coated PBA through a simple room‐temperature process (Figure 9a) [111]. The conformal nanoscale coating not only protected the surface from undesirable side reactions and Mn dissolution but also acted as a mechanical buffer against anisotropic volume changes, thereby mitigating intergranular cracking and enhancing structural stability during cycling. Beyond protective inorganic coatings, conductive organic polymers such as polydopamine [112], polyaniline [113], and polypyrrole [114, 115] are widely employed due to their compatibility with chemical polymerization and liquid−phase precipitation routes used in PBA synthesis. In situ polymer coating has been particularly effective, wherein monomer polymerization is triggered either by added oxidizers (e.g., ammonium persulfate) or by oxidation through HS transition‐metal centers in the PBAs (Figure 9b). The low‐defect KHCF@PPy synthesized by Xue et al. exhibited excellent electrochemical stability [115], retaining 86% of its capacity after 500 cycles at 50 mA g^−1^ and 71% at 1 A g^−1^ relative to 100 mA g^−1^. The improvement was attributed to the flexible PPy layer, which effectively alleviated structural strain and suppressed Fe dissolution during cycling.

Beyond in situ polymer coatings or high‐temperature pyrolytic carbonization, various carbon‐based materials, including graphene, graphene oxide (GO), reduced graphene oxide (r‐GO), carbon nanotubes (CNTs), and Ketjen black, have been integrated with PBAs through mechanochemical or chemical approaches [49, 117, 119, 120, 121, 122, 123, 124]. For instance, Shen et al. and Sun et al. utilized ball‐milling to embed pre‐synthesized PBA particles into graphene matrices [123, 125], achieving notable performance enhancement due to the uniform dispersion and pulverization of nanosized PBAs within a conductive network. Careful optimization of rotation speed and milling duration is essential to balance dispersion uniformity and structural integrity. Liquid‐phase mixing offers a promising alternative, as it provides higher dispersion uniformity compared with solid‐state techniques. Introducing the carbon matrix into the precursor solution before PBA formation enhances the degree of precursor interaction and serves as a nucleation substrate for controlled crystal growth. As illustrated in Figure 9c, precursors can be effectively anchored between electronegative GO layers via electrostatic interactions with oxygen‐containing functional groups [116]. During acid‐induced precipitation, oxidized HS‐Fe^3+^ species spontaneously reduce GO to r‐GO, consistent with observations reported by Tang et al. [120]. Exploiting the intrinsic metallic conductivity of CNTs, You et al. fabricated a PB@CNT composite featuring a necklace‐like morphology in which PB particles were threaded along CNT frameworks (Figure 9d) [117]. This architecture ensured robust electrical contact between PB particles and the current collector, even at low temperatures, yielding a four‐orders‐of‐magnitude enhancement in conductivity over bare PB and achieving 85% capacity retention at −25°C with an energy density of 408 Wh kg^−1^ at 0.1 C. Similarly, Mao et al. synthesized a NaKMnHCF/3DNC composite by in situ growth of PBAs on hierarchically porous three‐dimensional (3D) N‐doped carbon networks (3DNC) (Figure 9e) [118]. The porous carbon framework effectively mitigated particle aggregation and provided additional pseudocapacitive charge storage, resulting in a specific capacity exceeding 200 mAh g^−1^. First‐principles calculations indicated that Na‐ion adsorption energy at the NaKMnHCF/3DNC interface was lower than that on bare PBA surfaces, while enhanced Fe 3d‐electron delocalization contributed to improved conductivity and Na‐storage stability. Nevertheless, liquid‐phase synthesis remains largely confined to self‐decomposition or high‐pressure hydro/solvothermal routes, which limit their scalability. Future work should therefore prioritize the development of low‐cost, scalable, and environmentally benign liquid‐phase approaches for the uniform fabrication of carbon‐based PBA composites.

## 5 Evaluation of PBAs as Practical Cathodes

The intrinsic relationship between the structure and properties of PBAs has been discussed in the preceding sections, highlighting their potential competitiveness as next‐generation cathode materials. Nevertheless, several critical factors must be considered to enable their practical application. First, as previously noted, reducing PBA particle size enhances electrochemical performance by increasing the electrolyte–electrode contact area and improving wettability. However, smaller particles require greater amounts of binder to ensure sufficient cohesion within electrode films, which may lead to excessive binder decomposition and reduced electrode integrity. Second, the commonly observed high crystal‐water content (>5 wt.%) in PBAs strongly influences their physical, chemical, and electrochemical behavior. Although PBAs remain stable under ambient conditions, the coordinated and lattice water can dissociate during cycling, resulting in irreversible extraction and structural degradation. Theoretical calculations revealed that the binding energy of water to the lattice decreases from 0.8 to 0.1 eV upon Na removal [59], corroborated by differential scanning calorimetry (DSC) of charged electrodes and a gradual phase transition from hydrated monoclinic to dehydrated rhombohedral structure after extended cycling [29, 45]. These results underscore the instability of the crystal water of PBAs in their charged state.

Therefore, a comprehensive understanding of the role of crystal water, along with effective dehydration techniques and safe storage practices, is essential to prevent rehydration and maintain long‐term stability. In this section, we summarize the influence of particle size on PBA performance in PIBs and SIBs, bridging the gap between fundamental studies and practical implementation. We further discuss the impact of crystal water, including dehydration mechanisms, moisture‐driven degradation pathways, and strategies for preventing rehydration following initial dehydration.

### 5.1 The Impact of the Particle Size of PBAs

**FIGURE 10:**
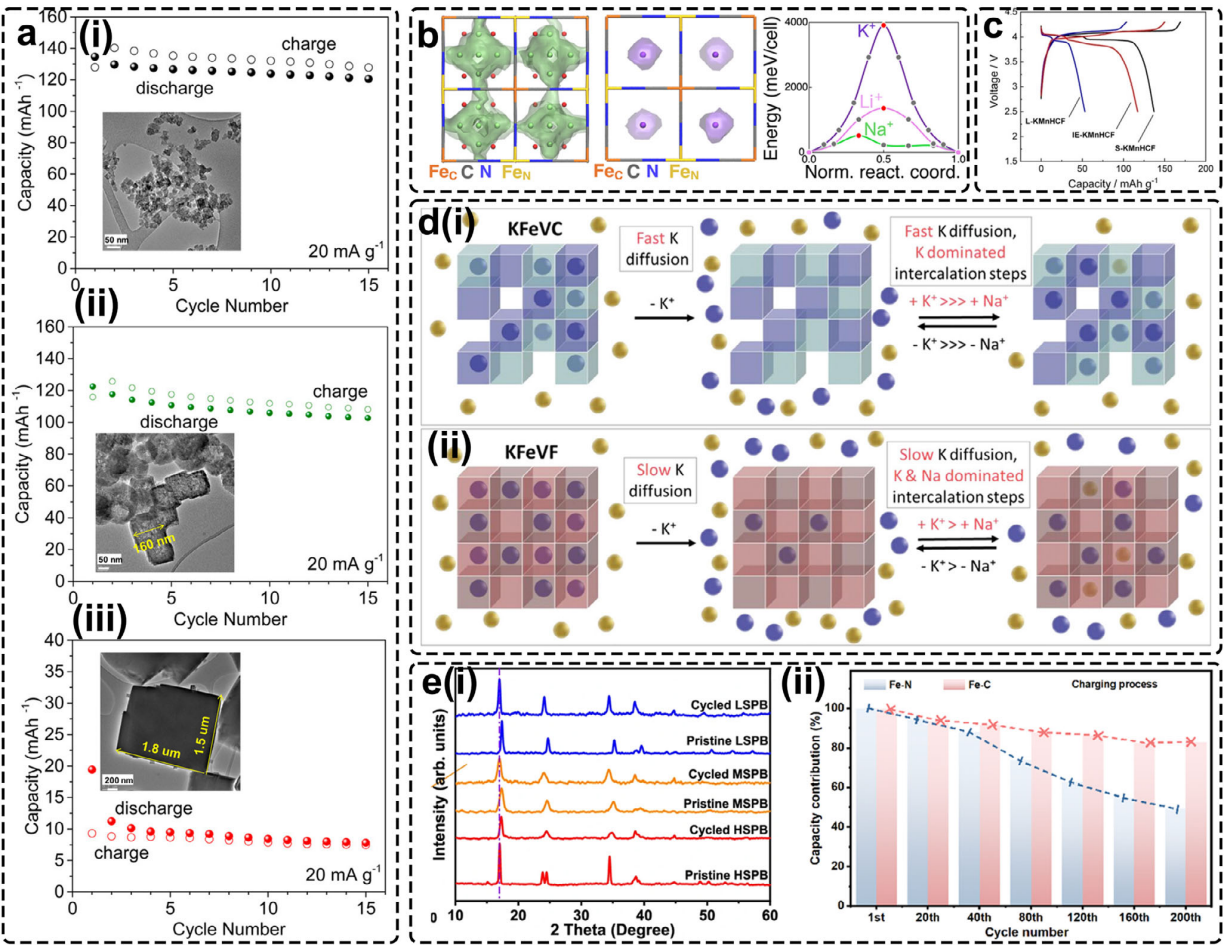
(a) The correlation between particle size and electrochemical performance of sizes (i) 50, (ii) 150 nm, and (iii) 1.5 µm at a current density of 20 mA g^−1^. Reproduced with permission [36]. Copyright 2017, American Chemical Society. (b) Trajectory densities at 300 K and Wyckoff occupation sites for Na (left) and K (middle); The potential energy profiles of Na and K‐ions diffusion (right). Reproduced with permission [126]. Copyright 2025, American Chemical Society. (c) Initial charge–discharge curves of S‐, L‐, and IE‐KMnHCFs. Reproduced with permission [64]. Copyright 2020, Wiley‐VCH. (d) Schematic illustration of cation (de)intercalation in the (i) anion vacancy‐containing KFeFe‐PBA framework and (ii) the anion vacancy‐free KFeFe‐PBA framework, when the KFeFe‐PBAs are used as cathode materials in NIBs. Reproduced with permission [127]. Copyright 2023, Wiley‐VCH. (e) (i) Laboratory‐based XRD patterns of pristine electrodes and electrodes after 100 cycles. (ii) The capacity contribution of HS‐Fe and LS‐Fe when discharged at different cycles. Reproduced with permission [128]. Copyright 2025, Springer Nature.

The pioneering work by He et al. demonstrated a strong correlation between particle size and the electrochemical performance of K‐based PBAs in non‐aqueous PIBs [36]. Three KFeHCF samples with distinct particle sizes were synthesized via a chelator‐assisted precipitation method: KFeHCF‐S (20 nm), KFeHCF‐M (200 nm), and KFeHCF‐L (> 1.5 µm) (Figure 10a,b). The study revealed that the electrochemical performance of K‐based PBAs is highly sensitive to particle size, with discharge capacity decreasing sharply from 140 to only 10 mAh g^−1^ as particle size increased from nanoscale to micrometer scale. This suggests that K^+^ diffusion becomes severely hindered in oversized PBAs due to extended transport pathways and limited ionic mobility. Complementary findings by Fiore et al. further indicated that capacity degradation in particles larger than ∼ 55 nm is primarily a kinetic limitation governed by reduced electronic and ionic conductivity [61]. Notably, the largest sample, KMF_297_, exhibited the most stable cycling behavior and the highest average Coulombic efficiency among the tested materials, implying that smaller particles, despite their improved kinetics, can promote excessive electrolyte decomposition at enlarged surface areas, ultimately accelerating capacity fading.

Ito et al. conducted a comparative *ab initio* study on the microscopic diffusion behaviors of Na^+^ and K^+^ ions in dehydrated AFe[Fe(CN)_6_] (A = Na or K) using density functional theory (DFT) calculations [126]. The results revealed that dehydrated NaFeHCF is an excellent Na^+^ conductor, exhibiting self‐diffusivities comparable to those of Li^+^. This behavior was attributed to the smaller tilting of [Fe(CN)_6_] octahedra during Na^+^ migration, which generates a shallower potential energy landscape **(left and middle panels,** Figure 10a). In contrast, larger K^+^ ions preferentially occupy body‐centered (8c) sites and show negligible diffusion between adjacent sites via face‐centered (24d) positions due to the high activation barrier associated with their larger ionic radius and stronger electronic interactions with the lattice **(right panel,** Figure 10b) [129]. Interestingly, K^+^ ions exhibited anisotropic diffusion along anion‐vacancy channels when structural defects were introduced, consistent with experimental observations that smaller, more defective particles display superior kinetics compared with larger, more ordered counterparts. Leveraging this insight, Hosaka et al. proposed that introducing an optimal number of vacancies can effectively enhance K^+^ mobility in large particles [64]. Specifically, incorporating ∼15% anionic vacancies via ion‐exchange enabled 1.5 µm KMnHCF particles to achieve comparable capacity to nanosized S‐KMnHCF, with a notably suppressed activation process relative to large L‐KMnHCF (Figure 10c). Building on this concept, our group recently demonstrated that K_1.49_Fe[Fe(CN)_6_]_0.8_, containing 20% anionic vacancies, can serve as an effective cathode for hybrid SIBs (Figure 10d) [127]. Atomic emission spectroscopy (AES) analysis confirmed that K^+^ insertion was promoted while Na^+^ intercalation was suppressed, even under Na‐concentrated electrolyte conditions. This selective K^+^ insertion led to increased intercalation voltage during discharge and enhanced long‐term cycling stability (72.3 mAh g^−1^ after 700 cycles at 100 mA g^−1^).

Currently, most reported K‐PBAs feature primary particle sizes below 100 nm to maximize their performance [32, 37, 39]. Future research should therefore focus on developing synthetic strategies that enable large‐particle K‐based PBAs to deliver comparable electrochemical behavior to their nanosized counterparts, acting as an essential step toward scalable electrode fabrication and industrial implementation.

Despite being excellent Na^+^ conductors at room temperature, the electrochemical capacity of Na‐based PBAs is less constrained by particle size than that of their K‐based counterparts. However, an upper performance limit still exists, as demonstrated by Zhang et al. [128]. Although a similar inverse correlation between particle size and capacity/rate performance was observed as in K‐PBAs, the study revealed that irreversible phase transitions, structural degradation, and deactivation of surface redox sites, accompanied by HS‐Fe dissolution, were the primary causes of long‐term capacity decay in large, Na‐rich, and low‐defect/crystal water HSPB particles (3–5 µm). These degradation processes led to significant morphological collapse (Figure 10e(i,ii)), indicating that capacity fading cannot be solely attributed to conventional factors such as defect density or crystal‐water content. To mitigate these issues, they implemented a dual‐regulation strategy involving Mn doping to tailor the local coordination environment of HS‐Fe [128], thereby alleviating internal stress during cycling. As a result, the optimized materials demonstrated enhanced cycling stability and robust electrochemical performance over a wide temperature range (−40°C to 100°C) when assembled into 18 650 and 33 140 cylindrical cells.

### 5.2 The Understanding of the Crystal Water in PBAs

**FIGURE 11:**
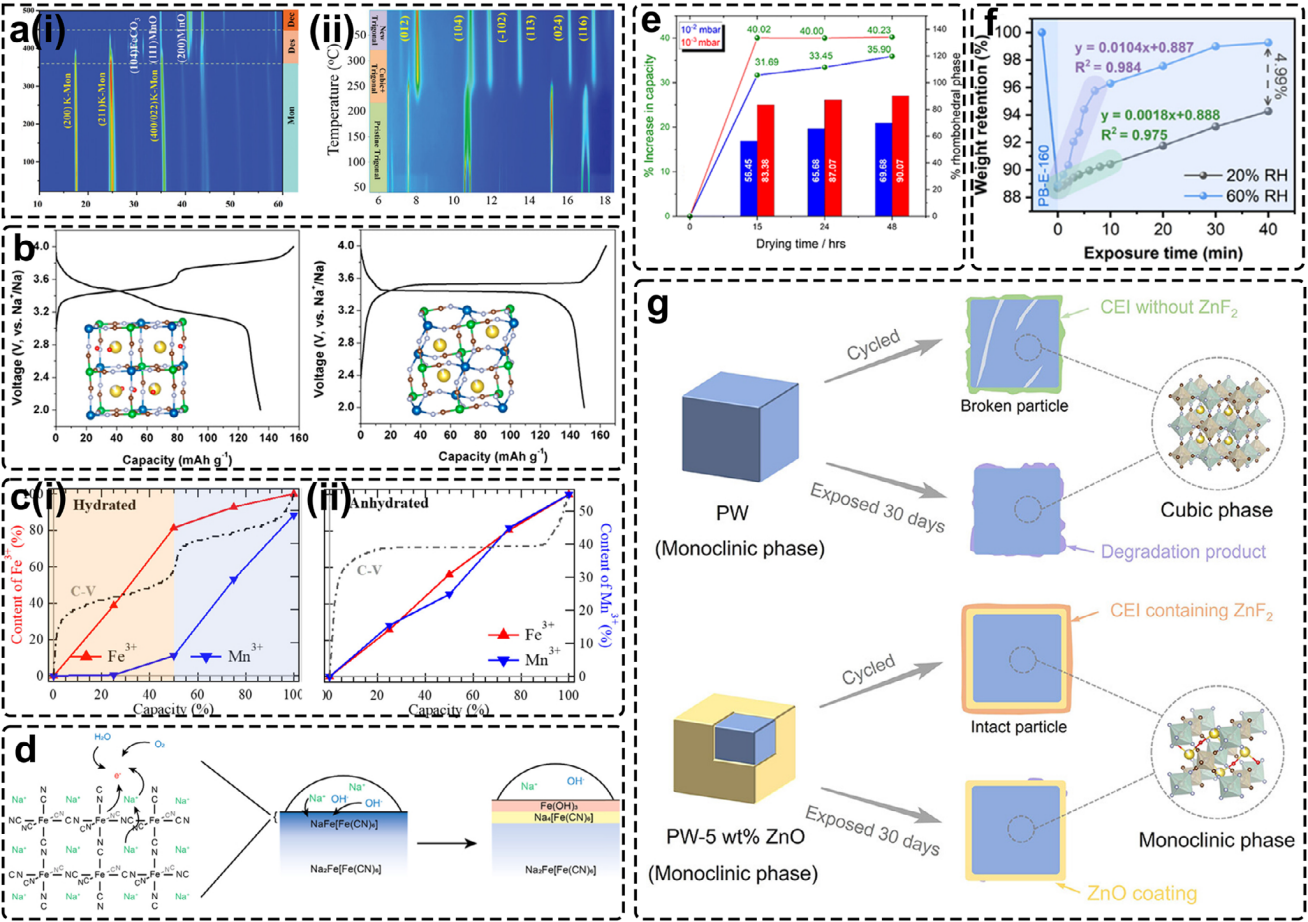
(a) 2D contour map of in situ high‐temperature synchrotron PXRD patterns of (i) the KFeHCF (ii) NaMnHCF from 40 to 400°C under Ar. Reproduced with permission [72, 130]. Copyright 2022, 2025, Wiley‐VCH. (b) The crystal structures and charge/discharge curves of (left) hydrated monoclinic and (right) dehydrated rhombohedral phases. Reproduced with permission [45]. Copyright 2015, American Chemical Society. (c) Concentration percentages of the oxidation states of Fe^3+^ and Mn^3+^ upon electrochemical potentials in the (i) hydrated samples and (ii) anhydrated samples. Reproduced with permission [131]. Copyright 2017, American Chemical Society. (d) Schematic illustration of the mechanism of Na loss and formation of Na_4_[Fe(CN)_6_]. Reproduced with permission [132]. Copyright 2021, American Chemical Society. (e) Comparative data of the percentage increase in capacity relative to the percentage of rhombohedral phase formed upon dehydration at 170°C for 15, 24, and 48 h using a dynamic vacuum of 10^−2^ and 10^−3^ mbar. Reproduced with permission [133]. Copyright 2023, Royal Society of Chemistry. (f) Mass variation of PB‐E‐160 under 60% RH and 20% RH. Reproduced with permission [134]. Copyright 2025, American Chemical Society. (g) Schematic illustration of the mechanism for the ZnO coating layer to improve the cycling performance and air stability of PW. Reproduced with permission [135]. Copyright 2024, American Chemical Society.

Crystal water markedly influences the physical, chemical, and electrochemical properties of PBAs, particularly for Na‐based PBAs. Owing to the strong preference of K^+^ ions for lattice incorporation, K‐based PBAs can be readily synthesized with near‐stoichiometric compositions, whereas researchers found it is trickier when K is replaced by smaller Na. Due to high crystalline perfection, K‐PBAs usually exhibit a monoclinic phase without any dehydration‐induced phase transition (Figure 11a(i)), while the initially Na‐rich monoclinic structure (*P2_1_/n*) transforms to rhombohedral (*R−3*) upon dehydration (Figure 11a(ii)), accompanied by unit‐cell shrinkage. The distinctive role of crystal water in stabilizing the Na‐PBA framework underscores the need for a deeper understanding of its structural flexibility and the resulting differences in electrochemical performance.

The dehydrated rhombohedral phase was first identified by Wang et al. and Song et al. in NaFeHCF and NaMnHCF, respectively [28, 45], following vacuum dehydration. As shown in Figure 11b(i,ii), two distinct redox plateaus observed in monoclinic NaMnHCF merged into a single plateau at 3.5 V after dehydration, accompanied by an increase in capacity from 137 to 150 mAh g^−1^. The rhombohedral phase exhibits a slightly smaller unit‐cell volume than the monoclinic counterpart due to greater rotation of the CN bond away from the Mn─N─C─Fe axis, caused by the displacement of Na^+^ ions toward the (111) direction. Wu et al. further investigated the valence‐state evolution of HS‐Mn and LS‐Fe in hydrated and dehydrated NaMnHCF using soft X‐ray absorption spectroscopy (XAS) [131]. In the hydrated sample, Fe and Mn redox processes occurred at distinct potentials, whereas their redox events overlapped in the dehydrated phase (Figure 11c(a‐b)), resulting in the convergence of two plateaus into one. The elevated Fe redox potential was attributed to enhanced ligand‐field stabilization energy (LFSE), which becomes comparable to the Fe^2+^/Fe^3+^‐Mn^2+^/Mn^3+^ ionization energy difference once the structural perturbation from crystal water is removed. Li et al. also reported that water‐free KMnHCF displayed delayed thermal runaway and reduced heat generation from HCN‐electrolyte reactions compared with its hydrated counterpart [74]. During electrochemical cycling, the removal of crystal water is irreversible as reported in several studies. For instance, Rudola et al. employed ex situ FTIR and observed the disappearance of the O─H stretching band at 3445 cm^−1^ after the first charge, which remained at low intensity upon subsequent discharge [29]. Li et al. further investigated the local Na environment during cycling using ^33^Na solid‐state NMR. They found that 50%–60% of Na^+^ ions resonate around 120 ppm in the fully discharged state [136], corresponding to a local environment similar to that of dehydrated NaMnHCF (D‐NaMnHCF). The release of crystal water into the battery system can trigger parasitic reactions with the electrolyte, generating by‐products that attack the SEI and ultimately lead to cell failure. Therefore, pouch cells fabricated by Wang et al. using fully dehydrated PBAs exhibited significantly less swelling and improved dehydration kinetics under vacuum drying [72, 73]. These findings highlight that water removal not only reduces processing complexity and cost but also enhances the thermal stability and safety of PBAs for practical cathode applications [128].

Despite the aforementioned advantages of anhydrous Na‐PBAs, several challenges remain in obtaining and stabilizing the dehydrated phase. First, dehydration kinetics are closely correlated with the crystal perfection of the synthesized PBAs, which depends on both the initial Na content and the vacancy concentration. Zhang et al. demonstrated that the dehydration transition temperature of NaFeHCF increases as the initial Na content decreases [128]; notably, Na‐poor LSPB underwent direct thermal decomposition without a phase transition. Similarly, a Na‐enriched R‐PB synthesized via NaI reduction yielded a fully rhombohedral phase upon drying, whereas the untreated R‐PB retained a mixed monoclinic‐rhombohedral structure [58]. A clear delay in dehydration was also observed in PBAs with higher vacancy levels [49, 137], underscoring the significance of synthetic control as discussed in Section 2.1. From a processing standpoint, complete dehydration of Na‐based PBAs typically requires temperatures exceeding 200°C under ambient pressure, increasing manufacturing costs and the likelihood of impurity formation (e.g., Fe_2_O_3_) [72, 134]. Furthermore, the dehydrated phase exhibits extreme sensitivity to moisture, reverting completely to the monoclinic phase within minutes of air exposure [29, 45]. Hence, maintaining dry or inert storage conditions prior to electrode slurry preparation is essential. Interestingly, Song et al. reported that the dehydration process is reversible and does not compromise electrochemical performance [45]. In their study, hydrated PBAs gradually transformed into the dehydrated phase after 10 electrochemical cycles, accompanied by diminished capacities of the monoclinic‐phase plateaus at 3.17 and 3.49 V. Conversely, Rudola et al. and Ojwang et al. observed significant capacity degradation in dehydrated PBAs following long‐term storage under humid conditions [29, 132]. Both rhombohedral and monoclinic samples transformed into Na‐poor cubic phases with ∼3% volume contraction after exposure to 75% relative humidity for seven days, resulting in irreversible capacity loss that could not be restored by subsequent drying. The proposed degradation mechanism (Figure 11d) proceeds as follows: (1) Na^+^ leaching via surface reactions with moisture forms NaOH, accompanied by spontaneous oxidation of HS‐Fe^2+^; (2) subsequent alkaline corrosion leads to irreversible formation of iron hydroxides and oxides; and (3) the oxidation process self‐terminates as a passivating layer forms [132].

Due to the high sensitivity and degradable nature of Na‐PBAs, particularly in their dehydrated form, careful handling is hence essential during both material preparation and subsequent electrode slurry processing. Maddar et al. demonstrated that Na‐PBAs can still be processed using environmentally friendly, water‐based slurries with CMC binders, followed by post‐drying to induce the rhombohedral phase [133]. The dehydration process was found to be kinetically controlled and strongly dependent on experimental conditions such as pressure, temperature, and duration. As shown in Figure 11e, rather than employing high temperatures (>200°C), reducing the pressure below 10^−3^ mbar proved more effective for complete water removal from composite Na‐PBA electrodes at moderate temperatures (150–170°C). Electrochemical evaluation revealed that the dehydrated PBAs exhibited higher capacity, reduced polarization within a narrower potential window, and improved Na^+^‐diffusion kinetics, features that may help suppress electrolyte decomposition at high voltages [133]. Wang et al. further quantified the optimal crystal‐water range as 2.86–4.28 wt%, corresponding to drying temperatures of 170–190°C [134]. Deviations from this range caused either excessive crystal defects (below threshold) or disappearance of the dehydrated phase (above threshold). The study also emphasized that storing dehydrated PBAs under low humidity (≈ 20%) significantly reduces water uptake rates compared with 60% humidity (Figure 11f), preserving performance and preventing safety risks such as cell swelling. To further improve air stability, a surface‐passivation strategy using acetate ligands was shown to effectively protect NaFeHCF against moisture degradation [138], a concept potentially extendable to other PBAs. Alternatively, Zhang et al. developed a mild vacuum‐drying approach that simultaneously formed a ZnO passivation layer on NaFeHCF [135]. This surface modification suppressed Na loss and irreversible degradation even after 30 days of humid‐air exposure. The aged PW‐5 wt% ZnO‐E retained 91.5% of its capacity after 200 cycles, significantly higher than the 64.5% retention of uncoated PW, matching a comparable performance of the fresh electrode. Beyond protection, the conductive ZnO layer also enabled stable high‐rate operation (80 mAh g^−1^ at 10 C) and promoted the formation of a robust ZnF_2_‐based CEI during cycling (Figure 11g). In addition to surface passivation, Lin et al. introduced trace Zr incorporation as a hydrophobic lattice‐engineering strategy to mitigate moisture‐induced degradation [139]. As a proof of concept, Zr‐doped PFHCF‐2 (1.6% Zr) maintained a high capacity of 144 mAh g^−1^ after one month of exposure to 45% humidity, showing minimal impurity formation and phase transition. The corresponding hard carbon (HC)||PFHCF‐2 full cell achieved 86% capacity retention after 550 cycles, outperforming the Zr‐free HC||FHCF cell, which retained only 65.9% after 400 cycles.

### 5.3 The Application of K−PBAs as the Cathodes of Hybrid SIBs

**FIGURE 12:**
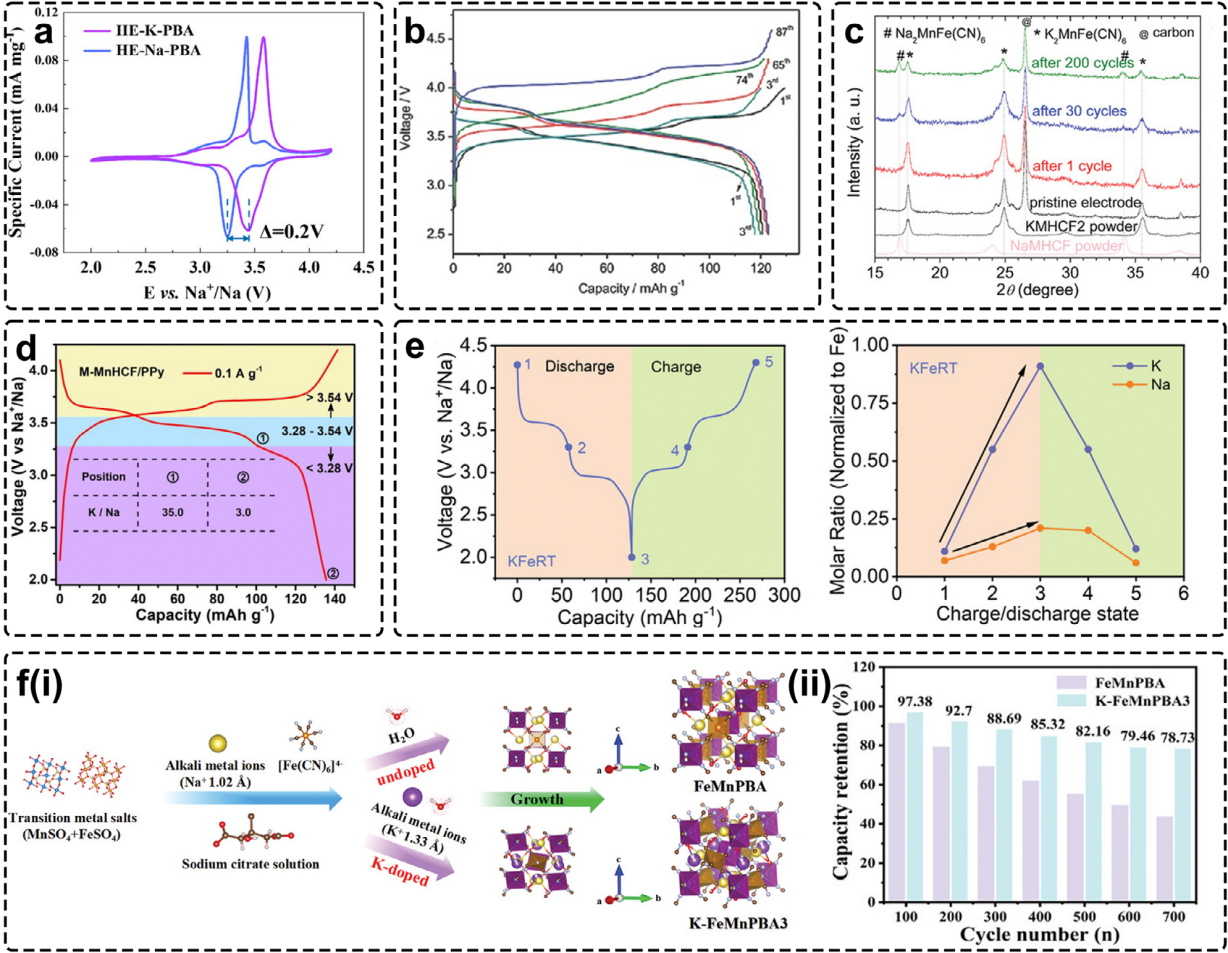
(a) First CV curves of HE‐K‐PBA and HE‐Na‐PBA at 0.05 mVs^−1^. Reproduced with permission [140]. Copyright 2023, American Chemical Society. (b) Charge/discharge curves and evolution with cycle number of working ion in a NaK/Na_2_MnFe(CN)_6_ cell with NaClO_4_ as the initial electrolyte salt. Reproduced with permission [141]. Copyright 2016, Wiley‐VCH. (c) XRD patterns of KMHCF electrodes before and after cycling (normalized with respect to Mn peak). Reproduced with permission [62]. Copyright 2019, Wiley‐VCH. (d) Charge and discharge curves of M‐MnHCF/PPy with inductively coupled plasma (ICP) tests at two different discharged states. Reproduced with permission [40]. Copyright 2024, Wiley‐VCH. (e) (i) Illustration of the different states of KFeRT during the first discharge process and the second charge processes; (ii) The Na and K contents (normalized to Fe) obtained from MP‐AES of KFeRT at the different states. Reproduced with permission [127]. Copyright 2023, Wiley‐VCH. (f) (i) Schematic illustration of the synthesis process of K‐doped and bare PBA samples; (ii) capacity retention of different cycles. Reproduced with permission [143]. Copyright 2024, Wiley‐VCH.

Owing to the structural flexibility and capability of accommodating multiple alkali cations within their lattices, K‐based PBAs have recently attracted significant attention as hybrid SIB cathodes. Benefiting from the intrinsic cation selectivity and the preferential intercalation of “size‐matched” larger cations, first−principles calculations revealed that the intercalation potential of the HS‐Fe^2+^/Fe^3+^ couple increases with cation size [129]. When Na^+^ is replaced by K^+^, the most thermodynamically stable intercalation site shifts from the face‐centered 24d to the body‐centered 8c position, resulting in a higher redox potential. This trend was experimentally verified by a 0.2 V difference between the redox plateaus of K‐ and Na‐based PBAs in SIB systems (Figure 12a) [140]. Xue et al. reported an electrochemically driven phase transition from NaMnHCF to KMnHCF when the former was used in an SIB employing a liquid Na–K alloy anode [141]. As shown in Figure 12b, both charge and discharge curves progressively shifted to higher voltages during cycling, while the dominant monoclinic phase of the K−PBA was retained even after 200 cycles (Figure 12c) [62]. The discharge potential for K‐ion storage exceeded 3.28 V, compared with < 3.28 V for Na‐ion storage (Figure 12d), confirming the preferential intercalation of K^+^ over Na^+^ during discharge. Building upon these findings, Dai et al. coated PBAs with an organic polymer layer to enhance electrical conductivity and, more importantly, to accelerate K^+^‐intercalation kinetics in MnHCF‐based cathodes operating via a competitive K^+^/Na^+^ insertion–extraction mechanism [40]. In our recent work, a defect‐engineering strategy was employed to further promote K^+^ intercalation [127]. The resulting K‐dominated intercalation behavior confirmed that the defective KFeRT framework exhibits strong selectivity toward K^+^ insertion throughout the entire voltage window (Figure 12e), explaining the higher intercalation potentials observed in this study relative to those of NaMnHCF and NaFeHCF cathodes reported previously [44, 142].

Beyond the increased intercalation potential and corresponding higher energy density, substituting Na^+^ with K^+^ fundamentally alters the structure and properties of PBAs. K‐based PBAs generally exhibit superior crystallinity, with markedly reduced defect density and lower crystal water content compared with Na‐based analogues synthesized under identical conditions. Liao et al. demonstrated that the total initial alkali‐cation ratio (Na + K)/Fe increased from 0.46 to 0.95 upon introducing K^+^ into Na‐based precursors [144], indicating that K^+^ ions are energetically more favorable for occupying interstitial sites within the PBA lattice and concurrently suppressing HS‐Fe oxidation. TGA by Zhang et al. further confirmed a substantial decrease in water content from 21 to 7.5 wt% with progressive K^+^ incorporation into NKPB‐0 (NaFeHCF) [145]. The presence of K^+^ also altered the preferred crystallographic orientation toward the (220) plane, enhancing capacity contributions from the HS‐Fe redox region (∼3.7 V). Owing to this optimized crystal structure and the synergistic influence of K^+^ within the framework, the resulting PBA achieved a high energy density of 450 Wh kg^−1^ and excellent reversibility with a stable capacity of 147 mAh g^−1^. Additionally, increasing K^+^ concentration was found to accelerate dehydration kinetics, reducing the dehydration onset temperature from 266 to 208°C and raising the thermal−decomposition temperature from 275°C (for NaMnHCF) to 340°C [130]. These changes collectively improve material safety by lowering the likelihood of HCN release during high‐temperature processing.

The larger ionic radius of K^+^ (1.33 Å) compared with Na^+^ (1.02 Å) can serve as a structural “pillar,” effectively widening Na^+^‐migration channels even at low substitution levels (Figure 12f(i)) [146]. Gao et al. optimized the K‐doping concentration to only 3% [143], achieving remarkable capacity retention of 85% (118 mAh g^−1^) at an ultrahigh rate of 30 C. Similarly, Zhao et al. electrochemically synthesized [K_0.444_Na_1.414_][Mn_3/4_Fe_5/4_](CN)_6_ [146], in which the residual 0.444 mol of K^+^ expanded the lattice and stabilized the framework. This K‐stabilized structure retained the integrity of the K‐PBA phase over 300 cycles rather than converting to the Na‐PBA phase, enabling highly reversible Na‐storage with a capacity of 128 mAh g^−1^ and 70% retention after 1800 cycles, significantly outperforming the K‐free FeMnPBA analogue (Figure 12f(ii)). Collectively, these findings highlight K substitution as a promising and distinctive strategy for enhancing Na‐PBA properties. Nevertheless, excessive K incorporation can introduce diffusion‐kinetic limitations similar to those observed in K‐PBAs, particularly when vacancy concentration is minimized, and K content becomes high [130]. As noted by Liu et al. [147], K‐PBAs generally exhibit superior cycling stability, whereas Na‐PBAs offer higher rate capability. Moreover, large K‐PBA particles (∼1.5 µm) typically require several hundred cycles for full activation [62]. Thus, optimizing synthetic parameters to balance the beneficial effects of K doping, such as improved crystallinity and elevated discharge potential, against its detrimental impact on rate performance remains essential. Future research should focus on carefully tuning the Na^+^/K^+^ ratio to achieve the optimal trade‐off between structural stability, energy density, and ion‐transport kinetics.

## 6 Summary and Outlook

**FIGURE 13:**
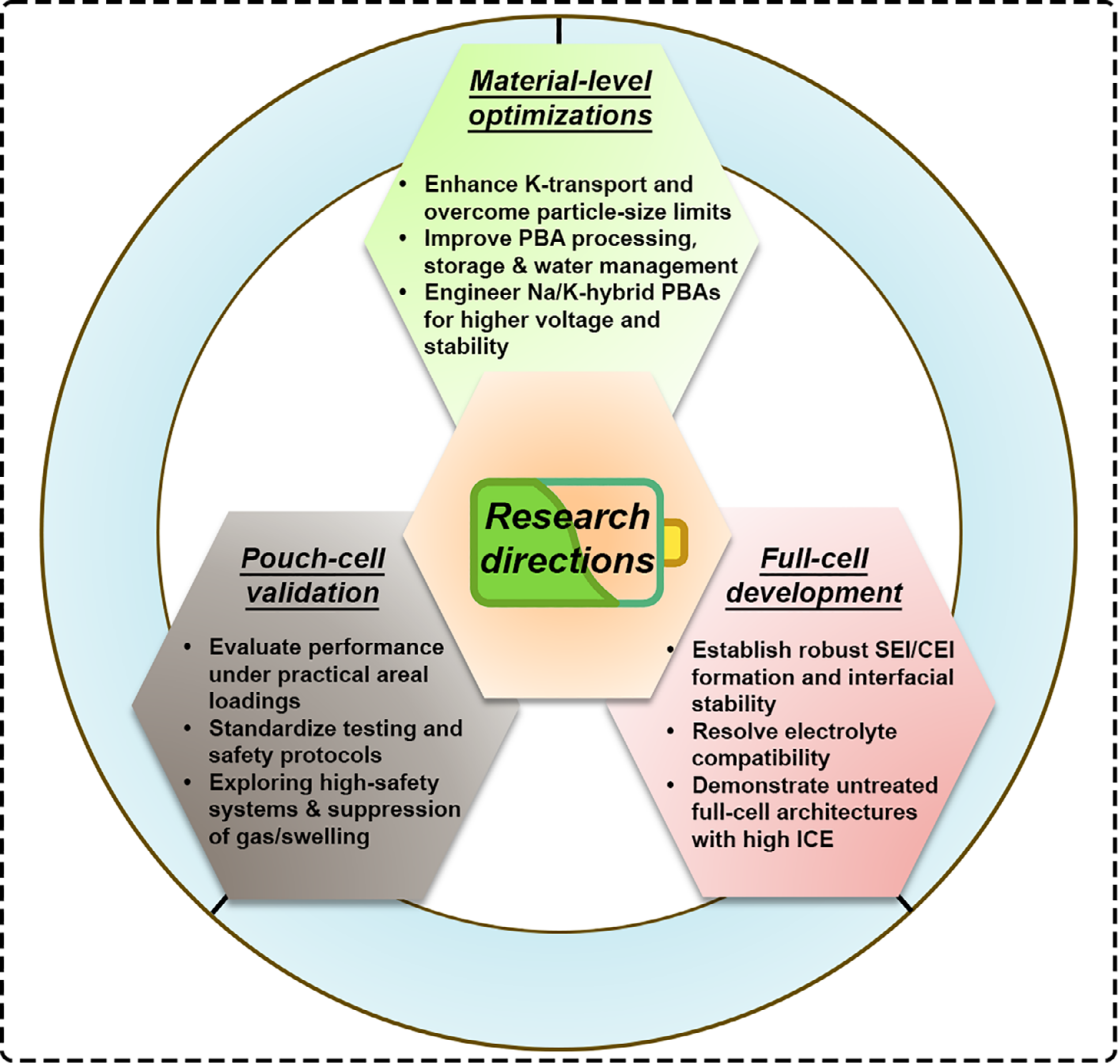
Future perspectives of development of non‐aqueous SIBs and PIBs.

PBAs represent a highly promising class of cathode materials for non‐aqueous rechargeable SIBs and PIBs. Their structural versatility, arising from the flexible coordination of alkali and transition‐metal cations, allows for diverse framework configurations with variable crystal distortions and lattice volumes—factors that fundamentally dictate their electrochemical properties, stability, and ion‐transport kinetics. Among the PBA family, FeHCF and MnHCF stand out for their optimal balance of energy density, cost‐effectiveness, and sustainability. Nevertheless, further improvements in cycling stability and rate performance are required to enhance their competitiveness for large‐scale applications. This review has provided a comprehensive overview of strategies for optimizing Na‐ and K‐based PBAs toward practical deployment, focusing on the control of crystallinity, morphology, and conductivity through facile synthesis routes. Slow and controlled nucleation and crystallization have been identified as the most effective approaches to achieving near‐stoichiometric PBAs with minimal defects. Such control can be realized via the judicious selection of chelating agents with tunable binding strength, concentration adjustment, the use of non‐aqueous solvents, or targeted post‐synthesis treatments. Morphological engineering can be achieved through chemical etching or the use of pre‐formed precursors with defined shapes, while the inherently poor conductivity of PBAs can be mitigated by in situ or ex situ conductive layer deposition, or compositing with carbonaceous matrices. In addition, recent advances elucidating the effects of particle size and crystal water have bridged fundamental understanding with practical design. The critical influence of particle size differs between systems: K‐PBAs possess a lower upper size limit than Na‐based analogues due to the poorer diffusivity of K^+^ within low‐defect lattices. Conversely, K‐based PBAs exhibit faster dehydration kinetics and enhanced thermal stability, effectively mitigating the adverse effects of crystal water, though these benefits must be carefully balanced in mixed K/Na systems. Despite these substantial advancements, several key challenges persist before PBAs can fully meet the performance demands of practical large‐scale energy‐storage applications. To address these challenges, continued exploration of viable solutions is essential to advance the commercial feasibility of enterprise‐scale PB and PBA materials for both SIBs and PIBs. Future research directions and strategies are summarized in Figure 13. Detailed analysis of these three major aspects will provide actionable insights for transitioning PBAs from laboratory research to market‐ready energy technologies.

### 6.1 PBA‐Synthesis

Vacancies in both Na‐ and K‐based PBAs remain poorly understood, particularly regarding their formation mechanisms and functional roles during electrochemical cycling. Further in‐depth investigation is needed to elucidate and control vacancy generation, especially for K‐PBAs employed in PIBs and hybrid SIBs. Future efforts should focus on developing synthetic routes capable of producing defect‐containing, large particle K‐PBAs to harness the beneficial effects of vacancies on rate performance and energy density. In contrast, the formation of dehydrated rhombohedral Na‐PBAs is highly sensitive to synthetic conditions, with conventional high‐pressure methods suffering from limited scalability. Hence, optimizing facile, low‐cost precipitation methods with minimal processing steps is necessary. Water removal from Na‐PBAs also poses a persistent challenge, as incomplete dehydration can lead to swelling and safety issues. Partial substitution of Na^+^ with K^+^ appears to be a promising approach, leveraging the synergistic structural effects of larger K^+^ ions. The optimal Na:K ratio should be carefully tuned to balance rate capability, cycling stability, crystallinity, water and defect levels, and particle size to maximize the achievable energy density and safety of practical SIBs.

### 6.2 Development of Full‐Cells

Despite these advances, the thermal stability of even the most robust K‐PBAs (< 250°C) remains considerably lower than that of layered metal oxide cathodes such as NMC used in LIBs [148]. Current studies primarily employ conventional Li/Na/KPF_6_‐carbonate electrolytes for comparison [74], yet KPF_6_‐based systems have been shown to be incompatible with stable SEI formation on PIB anodes such as graphite [149, 150]. Future work should therefore systematically evaluate the thermal‐runaway behavior of PBA full cells under varied electrolyte systems, considering both their reactivity and resulting SEI/CEI stability. In particular, nonflammable or concentrated K‐electrolytes merit investigation for their compatibility with both electrodes [151, 152]. Given the distinct decomposition pathways of APF_6_ (A = Li, Na, K) salts, their differing interfacial chemistries are expected to yield variable SEI compositions and thermal runaway thresholds.

From a practical standpoint, most current full‐cell demonstrations of K‐PBA||graphite configurations in PIBs rely on electrochemical pre‐potassiation of the graphite anode under half‐cell conditions before assembly, typically employing low active‐mass loadings and small particle PBAs (Table 3) [41, 153, 154, 155, 156]. Such pre‐potassiation steps may obscure the intrinsic material quality of PBAs and electrolyte stability, while also altering SEI formation and introducing unwanted side‐reaction by‐products [157, 158, 159]. Furthermore, reported energy densities are often calculated using the mass of the active electrode material alone, resulting in unrealistic performance metrics far below practical requirements. To facilitate accurate comparison and meaningful evaluation, future studies should adopt standardized reporting conventions, optimize the negative‐to‐positive capacity ratio (N/P), and increase material loading to > 5–10 mg cm^−2^. Maintaining an authentic SEI without reactive K metal is equally important for assessing the true commercial potential of PIB systems.

**TABLE 3:** Summary of the K‐PBA||graphite full‐cell performances in literature.

K−PBAs (particle size (nm))	Capacity (retention) (mAh g^−1^)	Voltage range@average (V)	Electrolytes	Energy density (Wh kg^−1^)	Capacity (Current Density (mAh g^−1^))	N/P ratios
K_1.75_Mn[Fe(CN)_6_]_0.93_ (20 – 30) [32]	80 (70%) at 30 mA g^−1^ after 60 cycles	*^1.5 – 4.5@3.50^*	*^0.7 M KPF6‐EC/DEC^*	*^536 (based on cathode half‐cell)^*	*^90 (100 mA g−1)^* *^62 (2000 mA g−1)^*	*^N/A^*
K_0.61_Fe[Fe(CN)_6_]_0.91_ (100) [37]	80 (96%) at 50 mA g^−1^ after 50 cycles (Graphite pre‐cycled)	*^2.0 – 4.0@3.10^*	*^0.8 M KPF6‐PC + 5% FEC^*	*^232 (based on whole cell system)^*	*^80 (50 mA g−1)^*	*^1.10^*
K_2_Mn[Fe(CN)_6_] (< 20) [160]	88.4 (85%) at 150 mA g^−1^ after 101 cycles (Electrolyte pre‐treated with K‐metal)	*^1.5 – 4.3@3.50^*	*^7 mol kg−1 KFSI‐DME^*	*^N/A^*	*^100 (150 mA g−1)^*	*^∼ 1.00^*
K_1.70_Mn[Fe(CN)_6_]_0.94_ (50 – 200) [161]	77 (80%) at 62.5 mA g^−1^ after 1000 cycles (Graphite fully pre‐discharged)	*^2.0 – 4.3@3.60^*	*^2.5 M KFSI‐TEP^*	*^260 (based on total mass of active materials)^*	*^120 (62.5 mA g−1)^* *^98 (375 mA g−1)^* *^60 (1250 mA g−1)^*	*^1.04^*
K_2_Mn[Fe(CN)_6_] (< 20) [162]	70 (75%) at 155 mA g^−1^ after 500 cycles	*^1.5 – 4.3@N/A^*	*^1 mol/kg K(PF6)0.75(FSI)0.25‐EC/PC^*	*^N/A^*	*^110 (15.5 mA g−1)^*	*^N/A^*
K_2_Fe[Fe(CN)_6_] (< 100) [163]	73 (95.8%) at 133 mA g^−1^ after 100 cycles (Graphite pre‐cycled)	*^2.0 – 4.0@3.1^*	*^2.76 M KFSI‐G1+TFTFE^*	*^138 (based on total mass of electrodes)^*	*^73 (133 mA g−1)^*	*^2.40^*
K_1.82_Mn[Fe(CN)6]_0.98_ (100) [93]	101 (< 99%) at 30 mA g^−1^ after 200 cycles (Graphite pre‐cycled)	*^1.3 – 4.2@N/A^*	*^2.9 M KFSI‐TEP^*	*^N/A^*	*^100 (30 mA g−1)^*	*^1.58^*
K_2_Mn[Fe(CN)6] (N/A) [156]	75 (90%) at 400 mA g^−1^ after 600 cycles (Graphite pre‐cycled)	*^2.0 – 4.0@3.00^*	*^0.91 M KFSI‐DEECl^*	*^N/A^*	*^91.6 (100 mA g−1)^* *^84.1 (200 mA g−1)^* *^61.2 (1000 mA g−1)^*	*^5.00^*
K_1.94_Mn[Fe(CN)6]_0.994_ (50 – 200) [33]	125 (98.5%) at 30 mA g^−1^ after 300 cycles (Graphite pre‐cycled)	*^1.5 – 4.2@3.58^*	*^2.5 M KFSI‐TEP^*	*^331.5 (based on total mass of electrodes)^*	*^125 (30 mA g−1)^* *^110 (300 mA g−1)^* *^80 (1000 mA g−1)^*	*^1.04^*
K_2_Mn[Fe(CN)_6_] (< 20) [164]	106 (∼ 100%) at 25 mA g^−1^ after 100 cycles	*^1.5 – 4.3@N/A^*	*^5.6 mol kg−1 KFSI‐G3^*	*^N/A^*	*^100 (25 mA g−1)^*	*^1.10^*
K_1.94_Mn[Fe(CN)6]_0.994_ (50 – 200) [42]	52 (71%) at 300 mA g^−1^ after 2000 cycles	*^1.5 – 4.3@3.61^*	*^1.5 M KFSI‐1,3−DX^*	*^316.5 (based on total mass of active materials)^*	*^125 (15.5 mA g−1)^* *^100 (50 mA g−1)^* *^70 (300 mA g−1)^*	*^0.92^*
K_1.98_Mn[Fe(CN)_6_]_0.98_ (30 – 40) [41]	101 (84.5%) at 750 mA g^−1^ after 3000 cycles (Graphite pre‐cycled)	*^1.5 – 4.2@3.6^*	*^3 M KFSI‐DME^*	*^326 (based on total mass of active materials)^*	*^150 (45 mA g−1)^* *^120 (750 mA g−1)^* *^75 (1500 mA g−1)^*	*^2.08^*

Despite recent progress, the development of K‐PBA||graphite full cells remains at an early stage compared with lithium‐based counterparts. Systematic studies on key formation parameters, such as the effects of varying N/P ratios, voltage cutoffs, electrolyte additives, and operating temperatures, are still limited. Each of these factors plays a critical role in determining the formation and stabilization of the SEI on graphite anodes. To obtain an accurate understanding of SEI evolution, detailed characterization should be performed under optimized full‐cell configurations that exclude metallic K, since the electrochemical and mechanical properties of SEIs formed in half cells often differ markedly from those formed in full cells without reactive potassium.

Several research groups have recognized and begun to investigate these discrepancies. Hosaka et al. employed gas chromatography–mass spectrometry (GC–MS) combined with density functional theory (DFT) calculations to elucidate the effects of electrolyte decomposition products formed during potassium metal soaking in KFSI and KFSI–KPF_6_ binary salt electrolytes [158]. They revealed that these decomposition products exert a positive influence on graphite anode performance, while oligo‐carbonates generated in KPF_6_‐based electrolytes detrimentally affect K‐PBA cathodes. Specifically, the improved performance in KFSI‐containing electrolytes was correlated with FSI‐derived decomposition anions, such as C_5_H_9_FNO_8_S_2_^−^. In contrast, solvent‐derived oligo‐carbonates from EC/DEC exhibit poor anodic stability and are readily oxidized at high cathode potentials, leading to increased irreversible capacity. Jeschull et al. compared SEI formation in half‐ and full‐cell configurations using a conventional KPF_6_‐EC/DEC electrolyte [157]. They observed that both systems produced an SEI composed of an inorganic KF‐rich inner layer and an organic‐rich outer layer after the first cycle; however, the SEI formed in the half cell was considerably thicker. This finding underscores the importance of studying SEI growth under realistic full‐cell conditions to avoid misleading interpretations derived from half‐cell tests. Looking forward, future studies should focus on advanced electrolyte formulations to better elucidate the coupled formation of both SEI and CEI. These emerging battery chemistries highlight the significant opportunities to better understand and tune the versatile electrochemistry of potassium‐ion systems, which may inspire novel battery designs that outperform the current state‐of‐the‐art potassium‐ion full‐cells.

In summary, the main issue of electrolyte compatibility in the full‐cell systems needs to be solved to push the development of PIBs forward, which can be divided into several aspects. First, the formation mechanism and charge‐transfer behavior of the SEI in full K‐ion batteries are still poorly understood, necessitating further systematic investigation. Second, greater effort is required to mitigate or control side reactions at high operating voltages. Although highly concentrated electrolytes can regulate polarization and suppress parasitic reactions through solvation effects, their oxidative stability at elevated potentials must be carefully evaluated. In addition, while KFSI‐based electrolytes have been extensively studied for their ability to form stable interfacial layers, their propensity to corrode current collectors under high‐voltage conditions should also be considered. Therefore, it is highly desirable to develop more suitable potassium electrolytes by optimizing K salts and/or solvent systems to simultaneously achieve stable interfacial chemistry while mitigating current collector corrosion.

### 6.3 Pouch‐Cell Development

From a practical standpoint, the key challenge for SIBs and PIBs is to translate their impressive material‐level energy densities into high cell‐level energy densities. To obtain meaningful guidance for developing high‐energy battery systems, it is essential to evaluate devices with realistic configurations. In fundamental laboratory research, their performance is typically assessed in coin‐type cells, which require minimal equipment, small amounts of active material, and simple fabrication procedures. However, the substantial differences in configuration between coin cells and practical devices limit their relevance in projecting real‐world performance. By contrast, pouch cells are increasingly regarded as suitable platforms to bridge laboratory studies and industrial implementation [165], owing to their compatibility with practical application and relatively straightforward fabrication.

However, the history of PBA‐based pouch cells in both sodium and potassium systems remains limited, as most studies still concentrate on PBA synthesis and half‐cell characterization. Pouch‐cell configurations were not reported until 2020, when NaFeHCF||hard‐carbon (HC) sodium pouch cells first appeared in literature [46]. A 0.5 Ah pouch cell was demonstrated, delivering ultra‐stable cycling with 78% capacity retention and a clear plateau at 2.9 V over 1000 cycles at 1 C. Using a larger‐scale 50 L reactor and leveraging the benefits of K incorporation into Na‐PBA [143], K‐FeMnHCF||HC achieved an energy density of 119.31 Wh kg^−1^ with an initial Coulombic efficiency of 88%. Nonetheless, most reported pouch‐cell data serve merely as demonstrations of cathode or electrolyte optimization strategies. The first formal investigation and performance evaluation of a PW pouch cell was reported in 2025 [166]. In this work, an aqueous processing route for HC anodes and PW cathodes was developed on a pilot line, where optimized dehydration protocols, electrode formulations, and calendaring yielded mechanically robust, defect‐free electrodes suitable for ≈1 Ah pouch cells [166]. The resulting HC∥PW pouch cells, assembled with industrially relevant areal loadings, exhibit stable cycling over a wide range of discharge rates and temperatures, with robust performance even at 10°C, while operation at 60°C leads to accelerated degradation attributed to SEI/electrolyte instability and localized Na plating [166]. Calendar‐life tests at different states of charge further confirm that capacity fade during prolonged storage remains limited, demonstrating that careful control of water exposure and electrode microstructure can reconcile the moisture sensitivity of PW with scalable, sustainable manufacturing of Na‐ion pouch cells. This study highlights how existing manufacturing facilities can be adapted for SIBs production and underscores the importance of addressing the lab‐industry gap.

In contrast, reports on K‐ion pouch cells remain scarce and lag behind those of Na systems. Most published studies employ non‐practical cathode mass loadings of only 1–2 mg cm^−2^, which result in low cell‐level energy, and many require pre‐potassiation prior to stacking [154, 155, 167]. To date, the highest reported mass loading for a K‐PBA cathode is 16.5 mg cm^−2^, corresponding to an areal capacity of 2.2 mAh cm^−2^ [41]. This high‐capacity pouch cell retained 80.7% of its capacity over 500 cycles at 3 C with a power density of 283 W kg^−1^, but it relied on PTFE binders [41], increasing electrode processing complexity. Using conventional slurry‐coated electrodes, the highest mass loading that affords balanced performance (consistent with particle size) is approximately 8 mg cm^−2^. These limitations further underscore the importance of fundamental material‐level optimization for enhancing the overall competitiveness of PIBs.

## Conflicts of Interest

The authors declare no conflicts of interest.

## Data Availability

The authors have nothing to report.
